# A New Simplified Coralmycin Derivative with Potent Anti-Gram-Negative Activity and Its Congeners Produced by the Myxobacterium *Corallococcus coralloides* M23

**DOI:** 10.4014/jmb.2605.05042

**Published:** 2026-10-19

**Authors:** Ha-Young Choi, Duc Dat Le, Bo-Min Kim, Won-Gon Kim

**Affiliations:** Infectious Disease Research Center, Korea Research Institute of Bioscience and Biotechnology, Yusong, Daejeon 305-806, Republic of Korea

**Keywords:** *Corallococcus coralloides*, Coralmycin, Structure-activity relationship, Gram-negative pathogens, DNA gyrase

## Abstract

Multidrug-resistant (MDR) Gram-negative bacterial infections urgently require new antibacterial agents with novel chemical scaffolds. In our continued investigation of minor metabolites produced by the myxobacterium *Corallococcus coralloides* M23, we isolated three new coralmycin derivatives, coralmycins J (1), K (2), and L (3), from large-scale fermentation cultures. Spectroscopic analyses revealed that these compounds represent structurally simplified derivatives of coralmycin with varying degrees of truncation. Compounds 1–4 (coralmycins J, K, L, and A) are new natural products isolated in our recent and current studies, while compound **5** (cystobactamid 919-2) is the externally reported reference compound used for comparison. Compound 1 exhibited potent antibacterial activity against both Gram-positive and Gram-negative bacteria and effectively inhibited *E. coli* DNA gyrase, whereas compounds 2 and 3 showed no detectable biological activity. Structure-activity relationship studies suggested that the *β*-methoxyasparagine moiety, ring C, or both, are important for antibacterial activity and DNA gyrase inhibition, whereas rings A and B present in compounds 4 and 5 appear to be dispensable. These findings provide valuable insights into the structural simplification of hexapeptidic coralmycins for the development of therapeutic agents against multidrug-resistant Gram-negative bacterial infections.

## Introduction

The growing emergence and spread of multidrug-resistant (MDR) bacterial pathogens pose a major challenge to global public health [1, 2]. Among them, MDR Gram-negative bacteria are particularly difficult to manage because carbapenems, which have long been regarded as one of the last-resort antibiotics for severe Gram-negative infections, are increasingly compromised by the global emergence of carbapenem-resistant strains, leaving few effective therapeutic options [3, 4]. Among the ESKAPE pathogens, the Gram-negative members (*Klebsiella pneumoniae*, *Acinetobacter baumannii*, *Pseudomonas aeruginosa*, and *Enterobacter* spp.) are of particular concern because of their multidrug resistance and limited treatment options [5, 6]. Owing to the critical threat they pose to public health, these pathogens have been classified by the World Health Organization (WHO) as priority targets for antibiotic discovery and development [7]. Accordingly, identifying new antibacterial agents effective against these resistant Gram-negative organisms remains a pressing need worldwide.

We recently reported the discovery of coralmycins A−I, a new group of antibacterial natural products, together with the known compound cystobactamid 919-2 (**5**), from fermentation cultures of the myxobacterium *Corallococcus coralloides* M23 [8, 9]. Coralmycin A (**4**) displayed potent antibacterial activity against both Gram-positive bacteria and clinically important Gram-negative pathogens. Structurally, compound **4** is a C-3 hydroxylated derivative of cystobactamid 919-2, which had previously been isolated from a myxobacterial strain belonging to the genus *Cystobacter* sp. by Muller’s group [10]. Notably, compound **4** displays 10-fold higher antibacterial activity than **5**. Both compounds function as DNA gyrase inhibitors [9, 10]. Additionally, both compound **4** and a derivative of compound **5** have demonstrated *in vivo* efficacy in mouse infection models infected with *Streptococcus pneumoniae* and *E. coli*, respectively [11, 12], highlighting their potential as promising antibiotic scaffolds. Structurally, compounds **4** and **5** have a hexapeptidic backbone consisting of three *para*-aminobenzoic acid units and two *para*-nitrobenzoic acid or *para*-aminobenzoic acid units, linked through an aspartic acid- or asparagine-derived moiety. However, because of their complex hexapeptidic scaffold, further optimization is required to improve their pharmacokinetic properties and facilitate large-scale production. More recently, Muller and co-workers reported the optimization of cystobactamids to enhance antibacterial activity [13] and improve pharmacokinetic profiles [14].

Through continued investigation of minor metabolites obtained from large-scale cultures of *Corallococcus coralloides* M23, we isolated a new simplified coralmycin derivative, coralmycin J (**1**), along with two related congeners, coralmycins K (**2**) and L (**3**) (Fig. 1). Herein, we describe the production, purification, structural characterization, and biological evaluation of compounds **1–3**, including their antibacterial properties and effects on *E. coli* DNA gyrase.

## Materials and Methods

### General Experimental Procedures

Optical rotation values were measured on a JASCO P-1020 polarimeter. Ultraviolet-visible (UV-Vis) spectra were recorded on a Shimadzu UV-1601 UV−visible spectrophotometer, and infrared (IR) spectra were collected using a Bruker EQUINOX 55 spectrometer. NMR experiments were performed on Bruker Biospin Avance spectrometers operating at 500, 700, or 900 MHz at the Korea Basic Science Institute. High-resolution electrospray ionization mass spectrometry (HRESIMS) data were acquired with a JEOL JMS-HX110/110A mass spectrometer.

### Fermentation and Isolation

Large-scale fermentation was conducted in a 5000-L fermenter operated at a 3000-L working volume. The inoculum was prepared through successive scale-up steps in 500-mL Erlenmeyer flasks, followed by 5-L, 50-L, and 500-L seed fermenters (Supplementary Information). Upon completion of the fermentation, the adsorbent resin and cells were harvested and extracted twice with acetone. After removal of the solvent under reduced pressure, the remaining aqueous phase was partitioned successively with chloroform and ethyl acetate. The ethyl acetate-soluble fraction was chromatographed on a Sephadex LH-20 column using methanol as the eluent, affording fractions containing compounds **4** and **5** as identified by TLC analysis. These fractions were subsequently subjected to preparative TLC on silica gel 60 F_254_ plates (Merck No. 1.05715.0001, Darmstadt, Germany) developed with CHCl_3_:MeOH (5:1) supplemented with 0.01% TFA. This process yielded four distinct bands, including two major bands, bands I (*R_f_* = 0.12) and II (*R_f_* = 0.48), and two minor bands, bands III (*R_f_* = 0.10) and IV (*R_f_* = 0.18). Further purification of band I by RP-18 TLC on silica gel 60 RP-18 F_254_ plates (Merck No. 1.15389.0001) using CH_3_CN:H_2_O (55:45) containing 0.01% TFA afforded compound **4** (*R_f_* = 0.50, 34.6 mg) together with an additional band, band I-1 (*R_f_* = 0.75). Compound **5** (717 mg) was isolated from band II following the same purification procedure. Subsequent RP-18 TLC of band I-1 with CH_3_CN:H_2_O (50:50) containing 0.01% TFA yielded compound **2** (*R_f_* = 0.57, 9.6 mg). Band III was subjected to sequential purification on silica gel and RP-18 plates with CHCl_3_:MeOH (5:1) and CH_3_CN:H_2_O (55:45), respectively, each containing 0.01% TFA, resulting in the isolation of compound **3** (*R_f_* = 0.39, 2.1 mg). Purification of band IV by RP-18 TLC with MeOH:H_2_O (70:30) containing 0.01% TFA furnished compound **1** (*R_f_* = 0.41, 0.8 mg).

Compounds **1—3** were assessed for purity by analytical HPLC using a YMC C18 column (4.6 × 150 mm, S-4 μm). Each compound showed a single peak at 220 nm using CH_3_CN:H_2_O (50:50) containing 0.01% TFA at a flow rate of 0.8 mL/min. The retention times of compounds **1, 2**, and **3** were 12.3, 3.3, and 3.9 min, respectively (Fig. S1).

The physicochemical properties of compounds **1—3** are as follows:

*Coralmycin J* (compound **1**): yellow powder; ${\left[\alpha \right]}_{D}^{25}$ = +16.1 (c 0.032, MeOH); UV (MeOH) λ_max_(log ε) 209 (4.50), 266 (sh) (4.19), 304 (4.29) nm; IR (KBr) ν_max_ 3357, 2924, 2850, 1660, 1604, 1521, 1508, 1309, 1138, 1095, 1022 cm^−1^; CD (MeOH) [θ]^25^ -912 (253), 830 (305) nm; HRESIMS *m/z* 550.1560 (M+H)^+^ (cald for C_26_H_24_N_5_O_9_, 550.1574).

*Coralmycin K* (compound **2**): yellow powder; ${\left[\alpha \right]}_{D}^{25}$ = +7.3 (*c* 0.032, MeOH); UV (MeOH) λ_max_(log ε) 229 (sh) (3.72), 260 (4.09), 291 (3.98) nm; IR (KBr) 3457, 3176, 2914, 2850, 1660, 1632, 1525, 1427, 1326, 1263, 1128 cm^−1^; CD (MeOH) [θ]^25^ -674 (227), 8.6 (317) nm; HRESIMS *m/z* 446.1179 (M+H)^+^ (cald for C_20_H_20_N_3_O_9_, 446.1199).

*Coralmycin L* (compound **3**): yellow powder; UV (MeOH) λ_max_(log ε) 229 (sh) (3.46), 266 (3.91), 291 (3.80) nm; IR (KBr) 3327, 2945, 2833, 1645, 1603, 1450, 1114, 1018 cm^−1^; HRESIMS *m/z* 286.0829 (M+H)^+^ (cald for C_14_H_12_N_3_O_4_, 286.0822).

### Determination of Antibacterial Susceptibility

Antibacterial activity was assessed using a broth microdilution assay according to a previously reported procedure [15]. Most bacterial strains were cultured to mid-logarithmic phase in Mueller-Hinton broth and subsequently diluted 1,000-fold in fresh medium. Cell suspensions were adjusted to a final density of 1 × 10^5^ cells/mL, and 0.2 mL aliquots were distributed into 96-well microtiter plates. *Acinetobacter baumannii* and *Streptococcus pneumoniae* were cultured in nutrient broth and Todd-Hewitt medium, respectively. Stock solutions of the test compounds and the reference antibiotics ciprofloxacin and nalidixic acid (Sigma) were dissolved in DMSO and added to the culture medium to give a final DMSO concentration of ≤ 0.05% (v/v). Wells containing 0.05% DMSO served as vehicle controls. Antibacterial activity was assessed by measuring minimum inhibitory concentrations (MICs) using two-fold serial dilution assays conducted in triplicate. Following incubation at 37°C for 24h, bacterial growth was monitored spectrophotometrically at 650 nm using a microplate reader. The MIC was defined as the lowest concentration that completely inhibited visible bacterial growth in all replicates.

### DNA Gyrase Assay

The effects of coralmycins on DNA gyrase-mediated DNA supercoiling were evaluated using a commercially available *E. coli* DNA gyrase assay kit (TopoGEN #TG2001G, USA). Test compounds, together with ciprofloxacin and nalidixic acid (Sigma), were prepared in DMSO and added to the assay mixture such that the final DMSO concentration remained below 0.05% (v/v). The reactions were performed in a total volume of 20 μL containing relaxed plasmid (0.5 μg), *E. coli* DNA gyrase (1 unit; approximately 20.5 nM), and 1 μL of the test compound. The reactions were incubated at 37°C for 30 min. The reactions were terminated by adding 10% (w/v) SDS (2 μL) followed by 10× DNA gel loading dye containing bromophenol blue (2 μL). The reaction mixtures were then extracted with chloroform:isoamyl alcohol (24:1, v/v), followed by vortexing and centrifugation. The upper aqueous phase was collected and subjected to electrophoresis on a 1% (w/v) agarose gel. DNA was visualized by staining with ethidium bromide. Due to the limited amounts of the purified natural products, DNA gyrase inhibition was evaluated using a concentration-dependent dose-response experiment with a single measurement at each concentration. Band intensities were quantified using ImageJ software (National Institutes of Health, USA), and IC_50_ values were determined by nonlinear regression analysis using GraphPad Prism version 8.0.2.

## Results and Discussion

### Structural Elucidation

Analysis of HRESIMS, ^1^H NMR, and ^13^C NMR data established the molecular formula of compound **1** as C_26_H_23_N_5_O_9_ (Figs. S2–S6 and Tables 1 and 2). Comparison of its ^1^H NMR spectrum with that of compound **5** revealed a close overall resemblance, except for the absence of resonances attributable to two isopropyl groups and the aromatic protons associated with rings A and B present in compound **5** (Fig. 1). These spectral differences indicated that compound **1** lacks rings A and B present in compound **5**. Comprehensive interpretation of the ^1^H-NMR, COSY, and HMQC spectra of compound **1** demonstrated the presence of two *para*-aminobenzoic acid units, one *para*-nitrobenzoic acid unit, and a *β*-methoxyasparagine unit. The connectivity of these structural units was confirmed by long-range HMBC correlations (Fig. 2). Thus, the planar structure of compound **1** was identified as a new derivative of compound **5** in which rings A and B are absent.

The relative stereochemistry of the *β*-methoxyasparagine unit in compound **1** was established through analysis of the ^3^*J*_HH_ coupling constant together with NOESY and selective NOE experiments (Table 2 and Figs. S7 and S8). The vicinal coupling constant of 8.1 Hz observed between H-2''' and H-3''' indicated an *anti* configuration between these protons. This assignment was further supported by key NOE correlations between H-3’’’ and NH-6’’’, and from H-2’’’ to OMe (Fig. 3), indicating that compound **1** had the same relative stereochemistry as compound **5**. In addition, the CD spectrum of compound **1** displayed Cotton effects [[*θ*]^25^(nm)(MeOH): -912 (253), 830 (305)] with closely matched those reported for compound **5** [[*θ*]^25^(nm)(MeOH): -4367 (251), 3999 (305)] [8] (Fig. S9). Collectively, these spectroscopic data established that compound **1** has the same configuration (S*R*) as compound **5**. The assignment of compound **1** as a natural metabolite rather than a degradation artifact is supported by the chemical stability of its aromatic peptide scaffold and by previous reports of naturally occurring truncated congeners within the coralmycin/cystobactamid family, including coralmycins E and F–I and cystobactamid 507 [9].

Interpretation of the HRESIMS and ^1^H and ^13^C NMR spectroscopic data established the molecular formula of compound **2** as C_20_H_19_N_3_O_9_ (Figs. S10–S13 and Tables 1 and 2). Its ^1^H NMR spectrum closely resembled that of compound **1**. The differences between the spectra of compounds **1** and **2** were as follows: the signals attributable to one *para*-aminobenzoic acid unit, including aromatic resonances at δ_H_ 7.97 (2H, d, 7.8) and 7.84 (2H, d, 7.8) and an amino proton signal at δ_H_ 10.57 (1H, s), were absent in compound **2**. In addition, the carboxamide NH_2_ resonances observed for compound **1** at δ_H_ 7.48 (1H, brs) and 7.56 (1H, brs) were no longer detected in compound **2**. Instead, an additional methoxy signal at δ_H_ 3.65 (3H, s) newly appeared in compound **2**. These spectral changes suggested that compound **2** lacks ring C and that the carboxamide NH_2_ group is replaced by a methoxy group. Key HMBC correlations supported the proposed substructure of compound **2**. In particular, the new methoxy resonance at δ_H_ 3.65 (4’’’-OMe) exhibited a long-range correlation with the ester carbonyl carbon at δ_C_ 170.8 (C-4’’’), indicating the presence of a methyl *β*-methoxyaspartic acid moiety (Fig. 2). Further analysis of the ^1^H-NMR and HMQC spectra of compound **2** revealed signals attributable to one *para*-aminobenzoic acid unit and one *para*-nitrobenzoic acid unit. The sequence and linkage of these fragments were verified by HMBC correlations (Fig. 2). Thus, the planar structure of compound **2** was assigned as a new methyl *β*-methoxyaspartic acid derivative of compound **1** in which ring C is absent.

The stereochemistry of the methyl *β*-methoxyaspartic acid residue in compound **2** was established from vicinal coupling and NOE analyses (Table 2 and Fig. S14). A small coupling constant of 4.0 Hz between H-2''' and H-3''' suggested a *gauche* orientation between these protons. This assignment was further supported by NOE correlations between H-2’’’ and H-3’’’ as well as between 3’’’-O-Me and NH-6’’’, indicating that compound **2** possesses a relative stereochemistry different from that of compound **1** (Fig. 3). In addition, the CD spectrum of compound **2** displayed Cotton effects [[θ]^25^(nm)(MeOH): -675 (227), 8.6 (317)] that were distinct from those observed for compound **1** (Fig. S9). Taken together, these spectroscopic data indicate that compound **2** possesses a stereochemical arrangement different from that of compound **1**. Accordingly, compound **2** was determined as a new methyl *β*-methoxyaspartic acid derivative lacking ring C and having a diastereoisomeric configuration of compound **1**. This stereochemical assignment is consistent with that reported for compound **6**.

High-resolution ESI mass spectrometry of compound **3** showed a protonated ion at *m/z* 286.0829 [M+H]^+^ (calcd. 286.0822 for C_14_H_12_N_3_O_4_), and analysis of the ^1^H and ^13^C NMR data led to the assignment of the molecular formula of C_14_H_11_N_3_O_4_ (Figs. S15–S18 and Tables 1 and 2). The ^1^H NMR spectrum of compound **3** closely resembled that of compound **2**. The differences between the spectra of compounds **2** and **3** were that signals attributable to the methyl *β*-methoxyaspartic acid residue of compound **2**, including an amide NH resonance at δ_H_ 8.43 (1H, d, 8.2), two methoxy proton signals at δ_H_ 3.65 and δ_H_ 3.33, and two methine resonances at δ_H_ 4.91 and δ_H_ 4.17, were absent in the spectrum of compound **3**. Instead, a broad amino signal appeared at δ_H_ 7.30 (2H, brs) in compound **3** (Table 1). These data suggested the absence of the methyl *β*-methoxyaspartic acid moiety in compound **3**. Further examination of the ^1^H-NMR and HMQC spectra indicated the presence of one *para*-aminobenzoic acid unit and one *para*-nitrobenzoic acid unit. In the HMBC spectrum (Fig. 2), the amino protons at δ_H_ 7.30 (1’’’’-NH_2_) showed a long-range correlation to the carbon at δ_C_ 129.9 (C-2’’’’), which in turn showed an HMBC correlation to the aromatic proton H-4’’’’(δ_H_ 7.86) of the *para*-aminobenzoic acid unit. Combined with the molecular formula, these data confirmed the absence of the methyl *β*-methoxyaspartic acid residue. Key HMBC correlations further established the linkage between the *para*-aminobenzoic acid and *para*-nitrobenzoic acid units (Fig. 2). Accordingly, compound **3** was assigned as a truncated derivative of compound **2** lacking the methyl *β*-methoxyaspartic acid moiety. Although this compound has previously been synthesized chemically and reported as 4-amino-*N*-(4-carbamoylphenyl)benzamide [16], the present work represents the first report of its isolation from a natural source.

### Effects of the New Derivatives on Bacterial Growth and *E. coli* DNA Gyrase Activity

Compounds **1–3** were assessed for their antibacterial activities against a panel of clinically relevant Gram-positive and Gram-negative pathogens. Their activities were compared with those of compounds **4** and **5**, as well as the reference antibiotic ciprofloxacin (Table 3). Because DNA gyrase is a highly conserved bacterial type II topoisomerase and has been established as the primary target of coralmycins and cystobactamids [9, 10], inhibition of *E. coli* DNA gyrase provides a representative assessment of target engagement for this class of compounds, although differences in whole-cell antibacterial activity among bacterial species may also reflect variations in membrane permeability and efflux mechanisms. Accordingly, the ability of compounds **1–3** to inhibit *E. coli* DNA gyrase-mediated DNA supercoiling was examined (Fig. 4 and Table 4). Ciprofloxacin and nalidixic acid were included as reference inhibitors in the enzyme assay. In control experiments, compounds **4** and **5** exhibited IC_50_ values of 0.05 and 0.99 μM, respectively, which were consistent with our previously reported values (0.08 and 0.95 μM, respectively) [9], supporting the reliability of the DNA gyrase inhibition data obtained in this study. Furthermore, compound **4** exhibited stronger antibacterial activity than compound **5**, consistent with our previous reports [8].

Compound **1**, a derivative of compound **4** lacking rings A and B, exhibited potent antibacterial activity against both Gram-positive bacteria (MICs = 0.03−0.5 μg/mL) and Gram-negative bacteria (MICs = 0.1−1 μg/mL) with activity comparable to that of compound **4**. Consistent with its antibacterial activity, compound **1** strongly inhibited *E. coli* DNA gyrase, with an IC_50_ of 0.09 μM, compared with 0.05 μM of compound **4**. Because these IC_50_ values were derived from single measurements at each concentration without statistical estimates of variability, the difference between the values should be interpreted as indicating a trend in relative inhibitory activity than a statistical significance difference. Together, these results indicate that removal of rings A and B does not abolish antibacterial activity or DNA gyrase inhibition, suggesting that rings A and B present in compound **4** are not essential for either activity.

In contrast, compound **2**, which lacks ring C and contains a methyl *β*-methoxyaspartic acid moiety with the diastereoisomeric configuration relative to the β-methoxyasparagine unit of compound **1**, showed no antibacterial activity up to 16 μg/mL. Consistent with this result, compound **2** did not inhibit *E. coli* DNA gyrase even at 300 μg/mL. Similarly, compound **3**, a derivative of compound **2** lacking the methyl *β*-methoxyaspartic acid moiety, exhibited neither antibacterial activity nor DNA gyrase inhibition at concentrations up to 32 and 30 μg/mL, respectively.

Because the hydroxyl group of the *β*-methoxyaspartic acid unit with the diastereoisomeric configuration was methylated in compound **2**, it remains unclear whether the complete loss of activity is primarily attributable to the absence of ring C, the replacement of the β-methoxyasparagine moiety, or a combination of both factors. Compound **6**, a *β*-methoxyaspartic acid derivative with the diastereoisomeric configuration of compound **4**, has previously been reported to exhibit antibacterial activity with MIC values of 1 and 4 μg/mL against MRSA CCARM 3167 and *E. coli* KCTC 1682, respectively, although its activity was 100-fold and 32-fold weaker than that of compound **4** [8]. Therefore, further studies will be needed to distinguish the individual contributions of ring C and the β-methoxyasparagine moiety to antibacterial activity and DNA gyrase inhibition.

Overall, this structure-activity relationship study provides a basis for the rational design and structural simplication of coralmycins and supports their further development as pharmaceutically viable antibacterial agents.

## Supplemental Materials

Supplementary data for this paper are available on-line only at http://jmb.or.kr.



## Figures and Tables

**Fig. 1:**
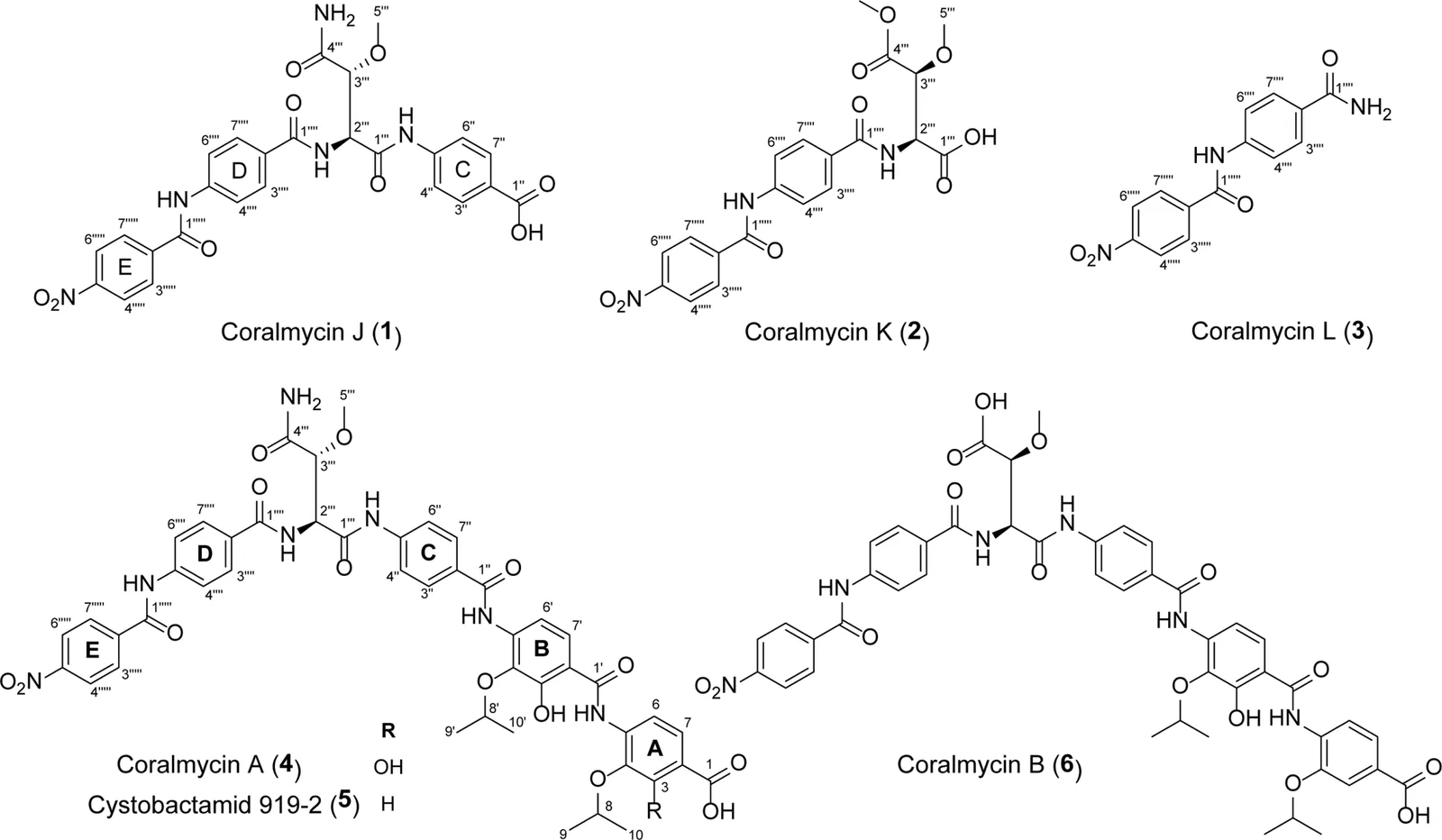
The structures of coralmycins J (1), K (2), and L (3), and related compounds.

**Fig. 2:**
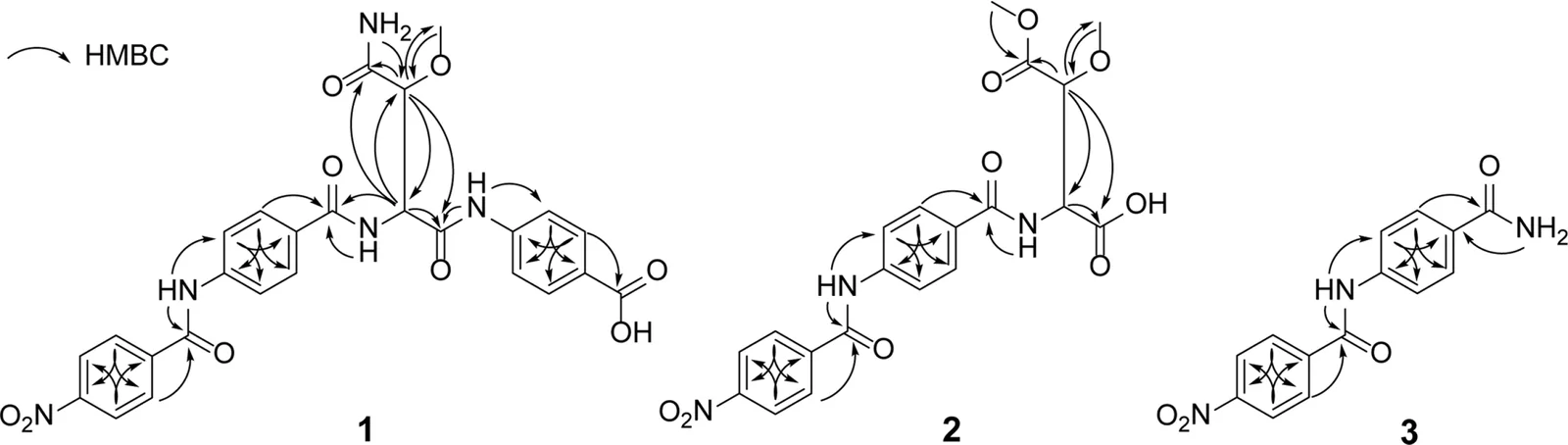
Key HMBC correlations of compounds 1-3.

**Fig. 3:**
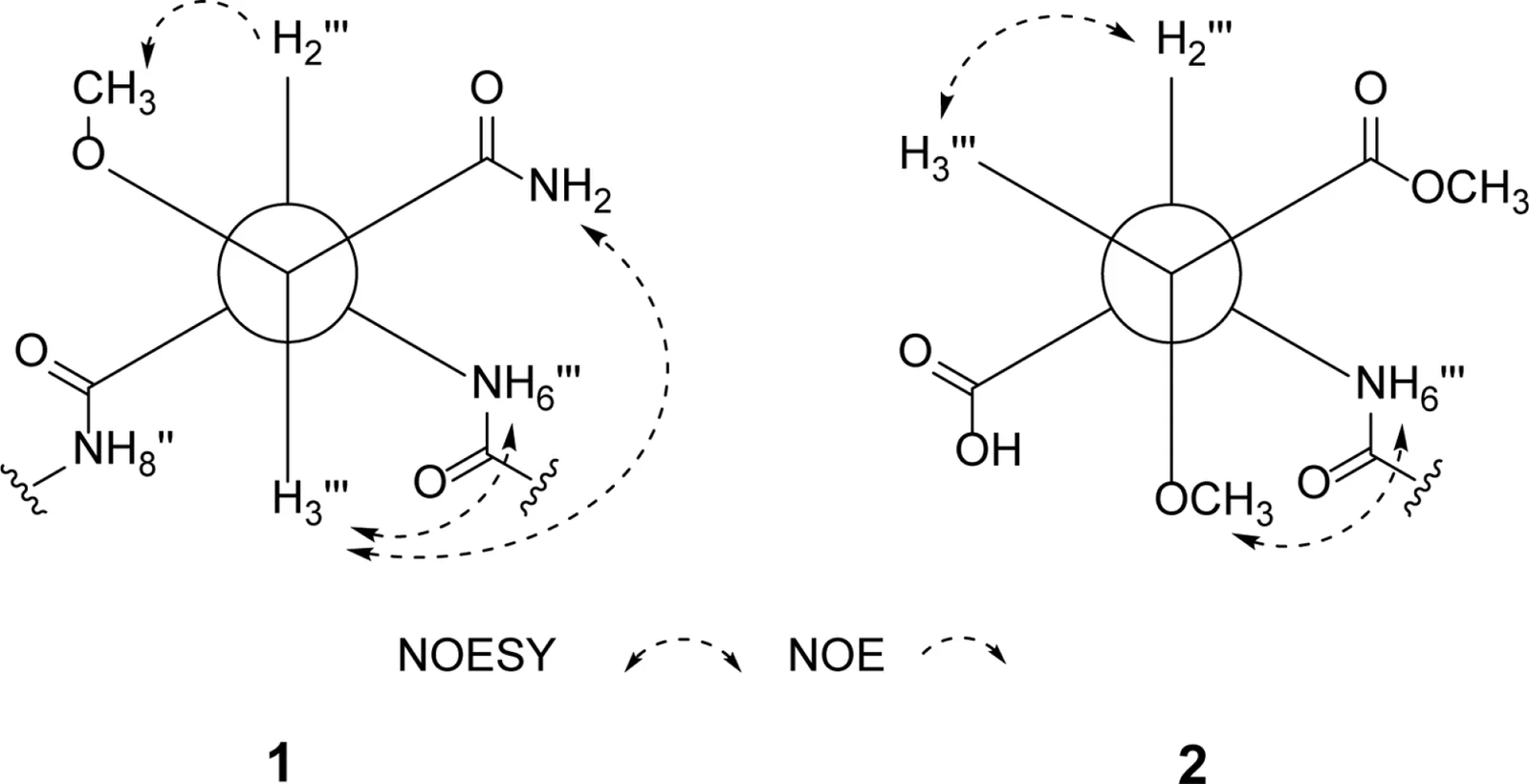
NOE correlations of compounds 1 and 2.

**Fig. 4:**
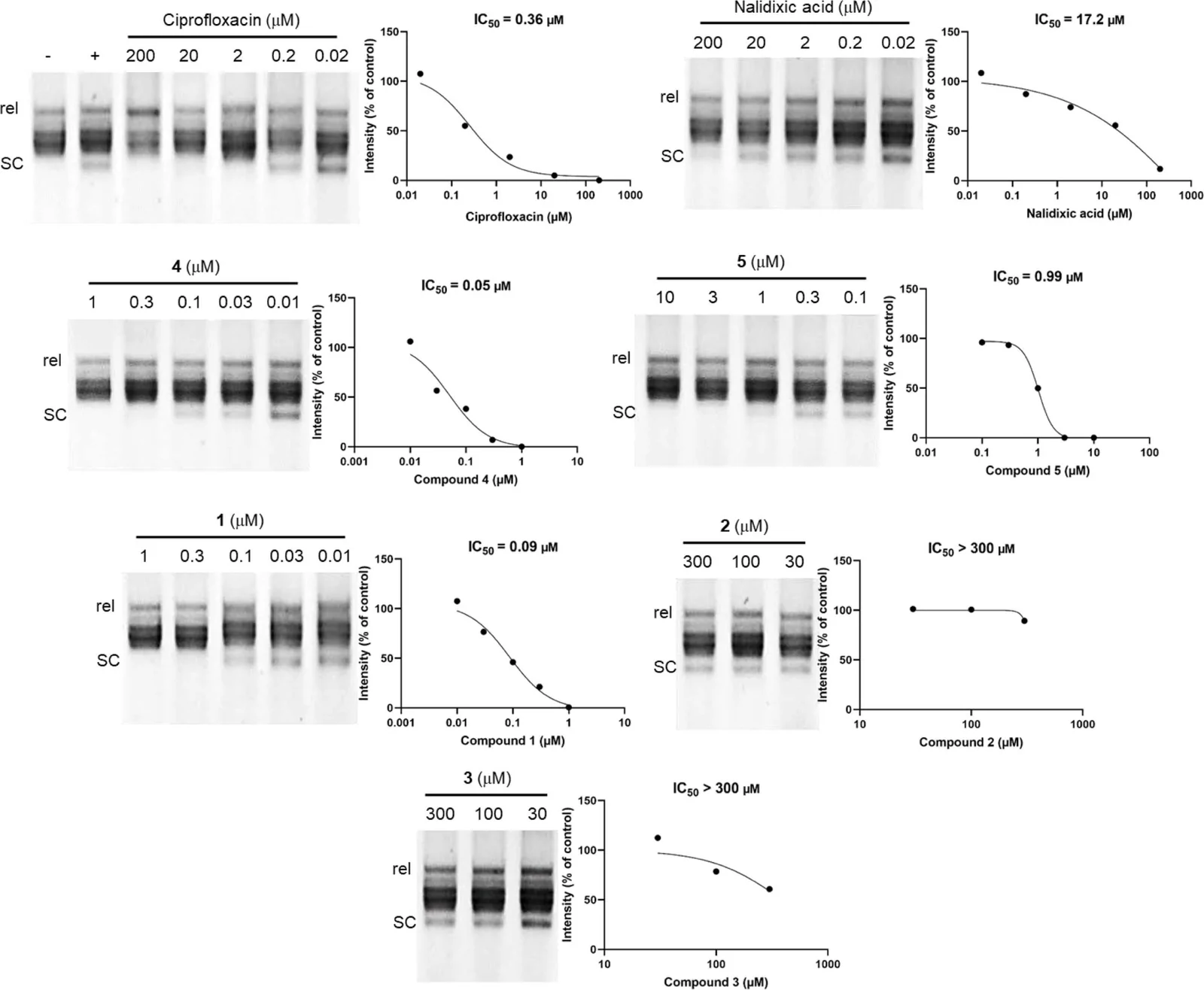
Effects of coralmycins J (1), K (2), L (3), and A (4), and cystobactamid 919-2 (5) on the DNA supercoiling activity of *E. coli* gyrase as visualized by agarose gel electrophoresis. Lane (-), reaction without *E. coli* gyrase; lane (+), gyrase reaction containing 5% DMSO; rel, relaxed plasmid; SC, supercoiled plasmid. Ciprofloxacin and nalidixic acid were included as reference inhibitors.

**Table 1:** ^1^H NMR spectroscopic data, δ (*J* in Hz), for compounds 1-3 in DMSO-*d*_6_.

Moiety	Position	**1** (700 MHz)	**2** (500 MHz)	**3** (900 MHz)	**5** (400 MHz)[8]
Ring A	1				
	2				
	3				7.56, brs
	4				
	5				
	6				8.50, d (8.3)
	7				7.58, d (8.3)
	8				4.75, m
	9,10				1.37, d (6.0)
	11-NH				10.97, brs
Ring B	1’				
	2’				
	3’				
	3’-OH				11.21, brs
	4’				
	5’				
	6’				7.50, d (8.5)
	7’				7.80, d (8.5)
	8’				4.32, m
	9’,10’				1.26, d (6.1)
	11’-NH				9.58, s
Ring C	1”				
	2”				
	3”,7”	7.97, d (7.8)			7.97, d (8.6)
	4”,6”	7.84, d (7.8)			7.83, d (8.6)
	5”				
	8”-NH	10.57, s			10.56, s
Asn-or Asp-derived unit	1’’’				
	2’’’	4.93, t (8.1)	4.91, dd (4.0, 8.2)		4.92, dd (8.0, 8.1)
	3’’’	4.10, d (8.1)	4.17**		4.09, d (8.0)
	4’’’-NH_2_	7.48, brs			7.47, brs
		7.56, brs			7.54, brs
	4’’’-OMe		3.65, s		
	5’’’	3.31, s	3.33, s		3.31, s
	6’’’-NH	8.48, d (8.1)	8.43, d (8.2)		8.46, d (8.1)
Ring D	1’’’’-NH_2_			7.30, brs	
	1’’’’				
	2’’’’				
	3’’’’,7’’’’	7.91*	7.81, d (8.7)	7.91, d (8.7)	7.90, d*
	4’’’’,6’’’’	7.91*	7.75, d (8.7)	7.86, d (8.7)	7.90, d*
	5’’’’				
	8’’’’-NH	10.82, s	10.8, brs	10.76, brs	10.8, s
Ring E	1’’’’’				
	2’’’’’				
	3’’’’’,7’’’’’	8.21, d (8.6)	8.04, d (8.6)	8.20, d (8.8)	8.21, d (8.6)
	4’’’’’,6’’’’’	8.39, d (8.6)	8.28, d (8.6)	8.38, d (8.8)	8.38, d (8.6)
	5’’’’’		-		-

*Overlapped signals. **Overlapped with the residual water signal. Chemical shifts (δ) are reported in ppm. Multiplicities are indicated as s (singlet), d (doublet), t (triplet), m (multiplet), brs (broad singlet), and dd (doublet of doublets).

**Table 2:** ^13^C NMR spectroscopic data, δ ppm, for compounds 1−3 in DMSO-*d*_6_.

Position	**1** (175 MHz)	**2** (125 MHz)	**3** (225 MHz)	**5** (100 MHz)[8]
1				166.9, C
2				125.7, C
3				113.9, CH
4				146.3, C
5				133.3, C
6				119.6, CH
7				122.6, CH
8				71.7, CH
9,10				21.6, CH_3_
1’				163.6, C
2’				116.4, C
3’				150.3, C
4’				138.4, C
5’				136.2, C
6’				115.3, CH
7’				124.9, CH
8’				75.6, CH
9’,10’				22.0, CH_3_
1”	164.9, C			164.3, C
2”	129.2, C			128.6, C
3”,7”	129.0, CH			128.4, CH
4”,6”	119.7, CH			118.8, CH
5”	143.0, C			142.1, C
1’’’	168.9, C	170.7, C		168.6, C
2’’’	55.8, CH	55.8, CH		55.7, CH
3’’’	80.3, CH	80.2, CH		79.7, CH
4’’’	171.2, C	170.8, C		170.8, C
4’’’-OMe		52.8, CH_3_		
5’’’	58.0, CH_3_	59.3, CH_3_		57.7, CH_3_
1’’’’	165.9, C	167.5, C	167.8, C	165.4, C
2’’’’	129.2, C	129.5, C	129.9, C	128.9, C
3’’’’,7’’’’	128.9, CH	129.3, CH	120.3, CH	128.2, CH
4’’’’,6’’’’	120.0, CH	120.8, CH	128.7, CH	119.6, CH
5’’’’	142.1, C	142.0, C	141.7, C	141.7, C
1’’’’’	164.7, C	165.8, C	164.8, C	164.2, C
2’’’’’	140.6, C	140.6, C	140.7, C	140.4, C
3’’’’’,7’’’’’	129.7, CH	129.8, CH	129.8, CH	129.3, CH
4’’’’’,6’’’’’	124.0, CH	124.1, CH	124.0, CH	123.5, CH
5’’’’’	149.7, C	149.1, C	149.6, C	149.2, C

**Table 3:** Antibacterial activities of 1–3 and related compounds.

Test organisms	MIC (μg/mL)					
	1	2	3	4	5	Cip*
*Staphylococcus aureus* RN 4220	0.03	>16	>32	0.03	0.125	0.125
MRSA CCARM 3167	0.125	>16	>32	0.25	0.5	2
QRSA CCARM 3505	0.5	>16	>32	0.25	1	128
*Streptococcus pneumonia* KCTC 5412	0.03	>16	>32	0.25	1	0.25
*Acinetobacter baumannii* KCTC 2508	0.125	>16	>32	0.125	2	0.25
*E. coli* KCTC 1682	0.5	>16	>32	0.25	2	0.06
*Pseudomonas aeruginosa* KCTC 2004	1	>16	>32	4	4	0.03

*Ciprofloxacin.

**Table 4:** Comparison of *E. coli* DNA gyrase inhibition (IC_50_, μM) and antibacterial activity (MIC, μg/mL) of compounds 1–3 and related compounds against *E. coli* KCTC 1682.

	1	2	3	4	5	Cip
IC_50_	0.09	>300	>300	0.05	0.99	0.36
MIC	0.5	>16	>32	0.25	2	0.06
