# Supplementary material for: A New Simplified Coralmycin Derivative with Potent Anti-Gram-Negative Activity and Its Congeners Produced by the Myxobacterium Corallococcus coralloides M23
Source: J Microbiol Biotechnol. 2026 Oct 19;36:e2605042. doi: 10.4014/jmb.2605.05042 (PMC13686806; doi:10.4014/jmb.2605.05042)
Supplement: Supplementary file 1 [file jmb-36-e2605042-supple.pdf]

Supplementary Information

A new simplified coralmycin derivative with potent anti-Gram-negative activity and its congeners produced by the myxobacterium *Corallococcus coralloides* M23

Ha-Yung Choi, Bo-Min Kim, and Won-Gon Kim*

Infectious Disease Research Center, Korea Research Institute of Bioscience and Biotechnology, Yusong, Daejeon 305-806, Korea

* Correspondence: wgkim@kribb.re.kr; Tel.: +82-42-860-4298

Fermentation. Fermentation was performed in CYS medium containing 0.5% casitone, 0.1% yeast extract, 0.3% soluble starch, 0.1% $MgSO_4 \cdot 7H_2O$, 0.05% $CaCl_2$, 50 mM 4-(2-hydroxyethyl)-1-piperazineethanesulfonic acid (HEPES), 0.4% trace element solution, and 0.5 μg/mL cyanocobalamin. The trace element solution contained 100 mg/L $MnCl_2 \cdot 4H_2O$, 20 mg/L $CoCl_2$, 10 mg/L $CuSO_4$, 10 mg/L $Na_2MoO_4 \cdot 2H_2O$, 20 mg/L $ZnCl_2$, 5 mg/L LiCl, 5 mg/L $SnCl_2 \cdot 2H_2O$, 10 mg/L H_3BO_3, 20 mg/L KBr, 20 mg/L KI, and 8 g/L EDTA Na-Fe^{3+} salt (trihydrate). A piece of agar from the mature plate culture of the producing strain was inoculated into a 500-mL Erlenmeyer flask containing 100 mL of sterile seed liquid medium (same composition as above) and cultured on a rotary shaker (150 rpm) at 28 °C for 3 days. Then, 300 mL of the seed culture was transferred into a 5-L Jar fermenter containing 2.5 L of the same medium containing 100 g of Amberlite XAD16 (Sigma, USA), and cultivated at 28 °C for 2 days with an aeration rate of 0.4 v/min and agitation at 350 rpm. The resultant 3 L culture was transferred into a 50-L fermenter containing 30 L of the same medium containing 1.2 kg of Amberlite XAD16, and then cultivated at 28 °C for 3 days with an aeration rate of 0.4 v/min and agitation rate of 200 rpm. The resultant 30 L culture was transferred into a 500-L fermenter containing 300 L of the above medium containing 12 kg of Amberlite XAD16, and then cultivated at 28 °C for 3 days with an aeration rate of 0.4 v/min and agitation at100 rpm. The resultant 300L culture was transferred into a 5000-L fermenter containing 3000 L of the same medium with 120 kg of Amberlite XAD16, and fermentation proceeded at 28 °C for 6 days with an aeration rate of 0.4 v/min and agitation at 70 rpm.

Legends for Figures

Figure S1. HPLC profiles of coralmycins (J (**1**), K (**2**), L (**3**), and A (**4**)) and cystobactamids 919-2 (**5**). 4
Figure S2. Positive HRESIMS spectrum of coralmycin J (**1**). 5
Figure S3. ^{1}H-NMR spectrum of coralmycin J (**1**) measured in DMSO-d_6 at 700 MHz. 6
Figure S4. COSY spectrum of coralmycin J (**1**) measured in DMSO-d_6 at 700 MHz. 7
Figure S5. HMQC spectrum of coralmycin J (**1**) measured in DMSO-d_6 at 700 MHz. 8
Figure S6. HMBC spectrum of coralmycin J (**1**) measured in DMSO-d_6 at 700 MHz. 9
Figure S7. NOESY spectrum of coralmycin J (**1**) measured in DMSO-d_6 at 700 MHz. 10
Figure S8. The NOE differential spectra of coralmycin J (**1**) measured in DMSO-d_6 at 700 MHz. 11
Figure S9. CD spectra of coralmycins A (**1**) and B (**2**) 12
Figure S10. Positive HRESIMS spectrum of coralmycin K (**2**). 13
Figure S11. ^{1}H-NMR spectrum of coralmycin K (**2**) measured in DMSO-d_6 at 700 MHz. 14
Figure S12. HMQC spectrum of coralmycin K (**2**) measured in DMSO-d_6 at 500 MHz. 15
Figure S13. HMBC spectrum of coralmycin K (**2**) measured in DMSO-d_6 at 500 MHz. 16
Figure S14. NOESY spectrum of coralmycin K (**2**) measured in DMSO-d_6 at 500 MHz. 17
Figure S15. Positive HRESIMS spectrum of coralmycin L (**3**). 18
Figure S16. ^{1}H-NMR spectrum of coralmycin L (**3**) measured in DMSO-d_6 at 500 MHz. 19
Figure S17. HMQC spectrum of coralmycin L (**3**) measured in DMSO-d_6 at 500 MHz. 20
Figure S18. HMBC spectrum of coralmycin L (**3**) measured in DMSO-d_6 at 500 MHz. 21

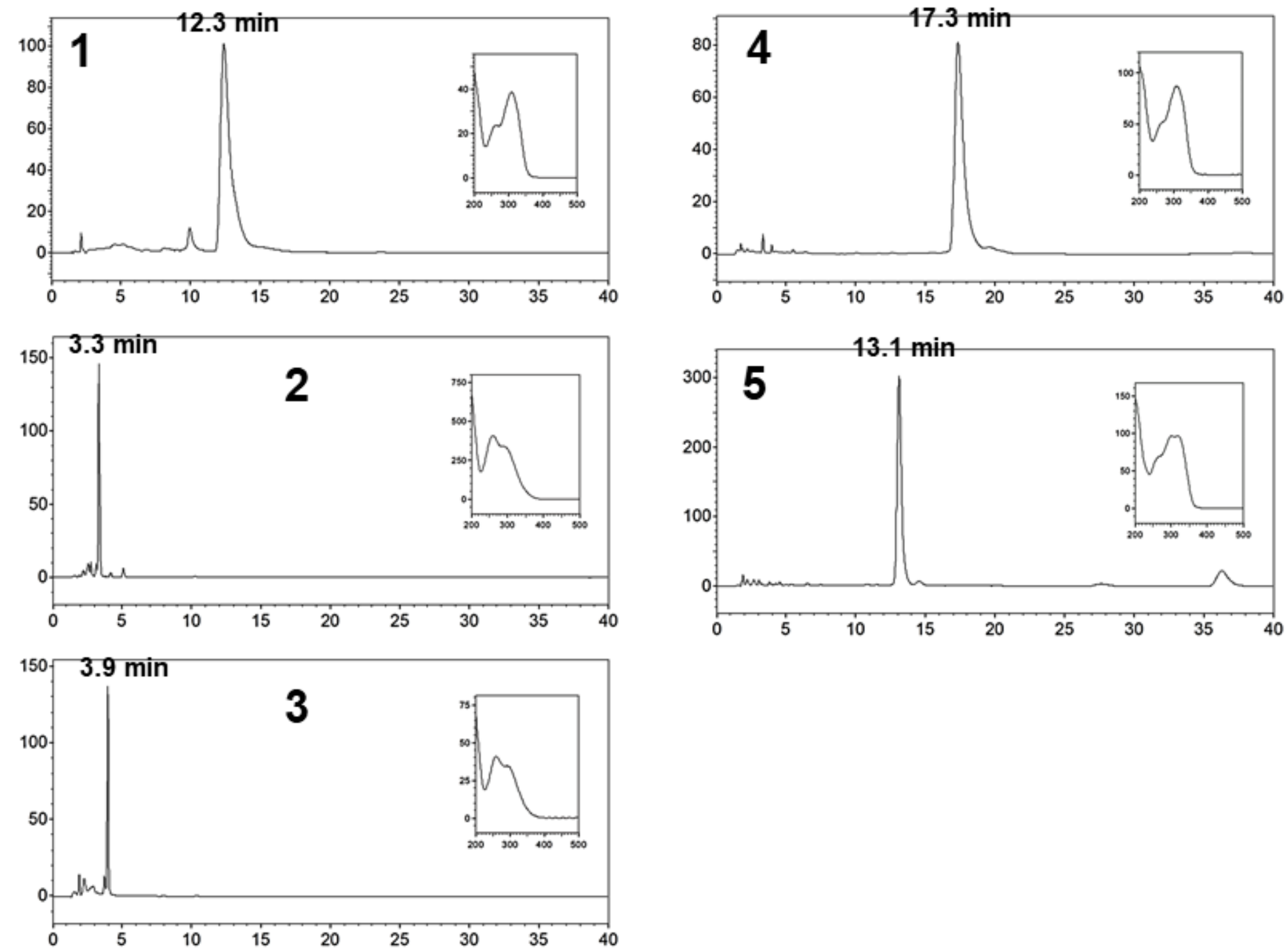

Figure S1. HPLC profiles of coralmycins (J (**1**), K (**2**), L (**3**), and A (**4**)) and cystobactamid 919-2 (**5**). J'sphere ODS-H80 (150x4.6 mm I.D), 50% ACN + 0.01% TFA, 0.8 mL/min, 25°C

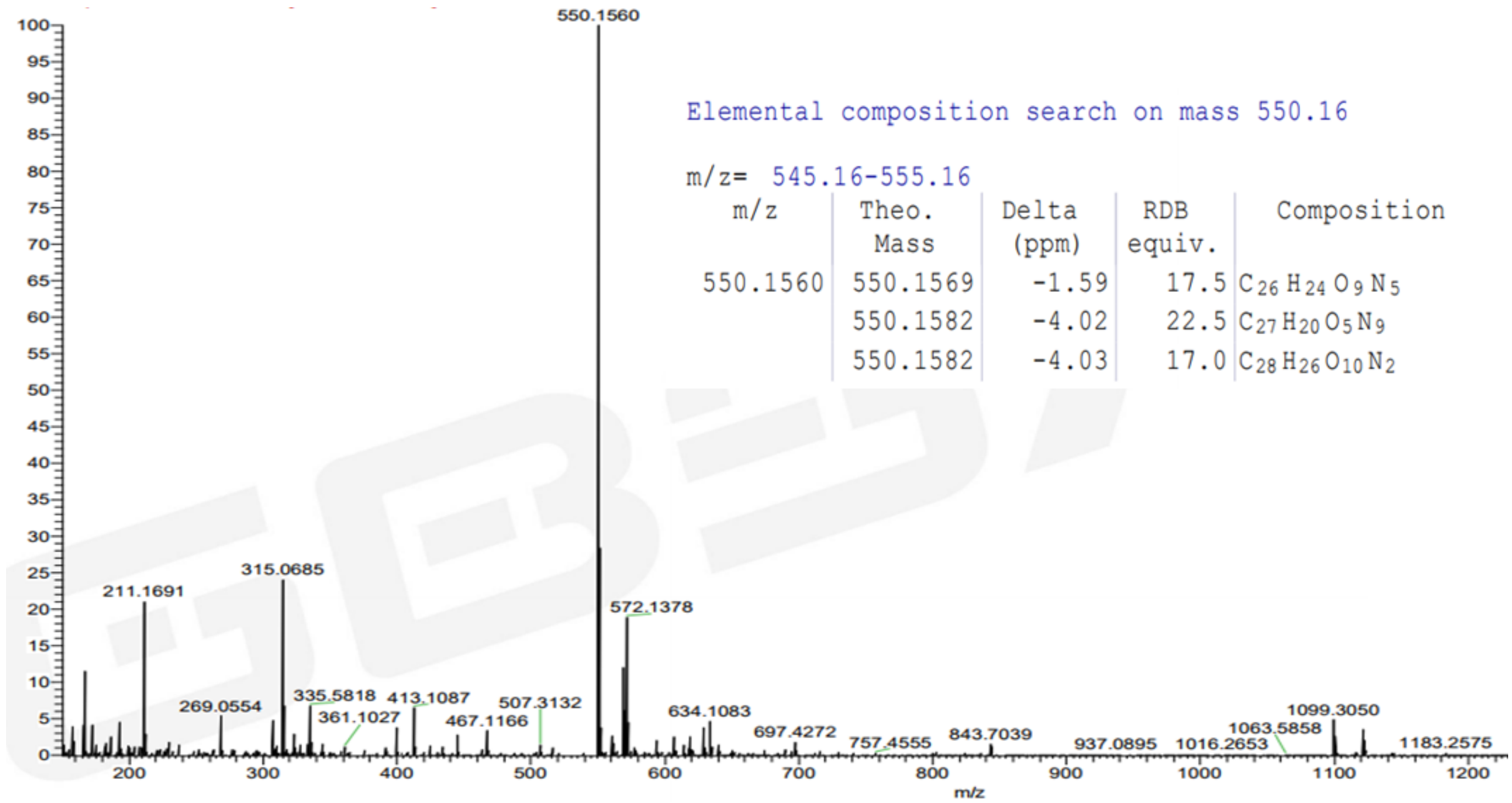

Figure S2. Positive HRESIMS spectrum of coralmycin J (**1**).

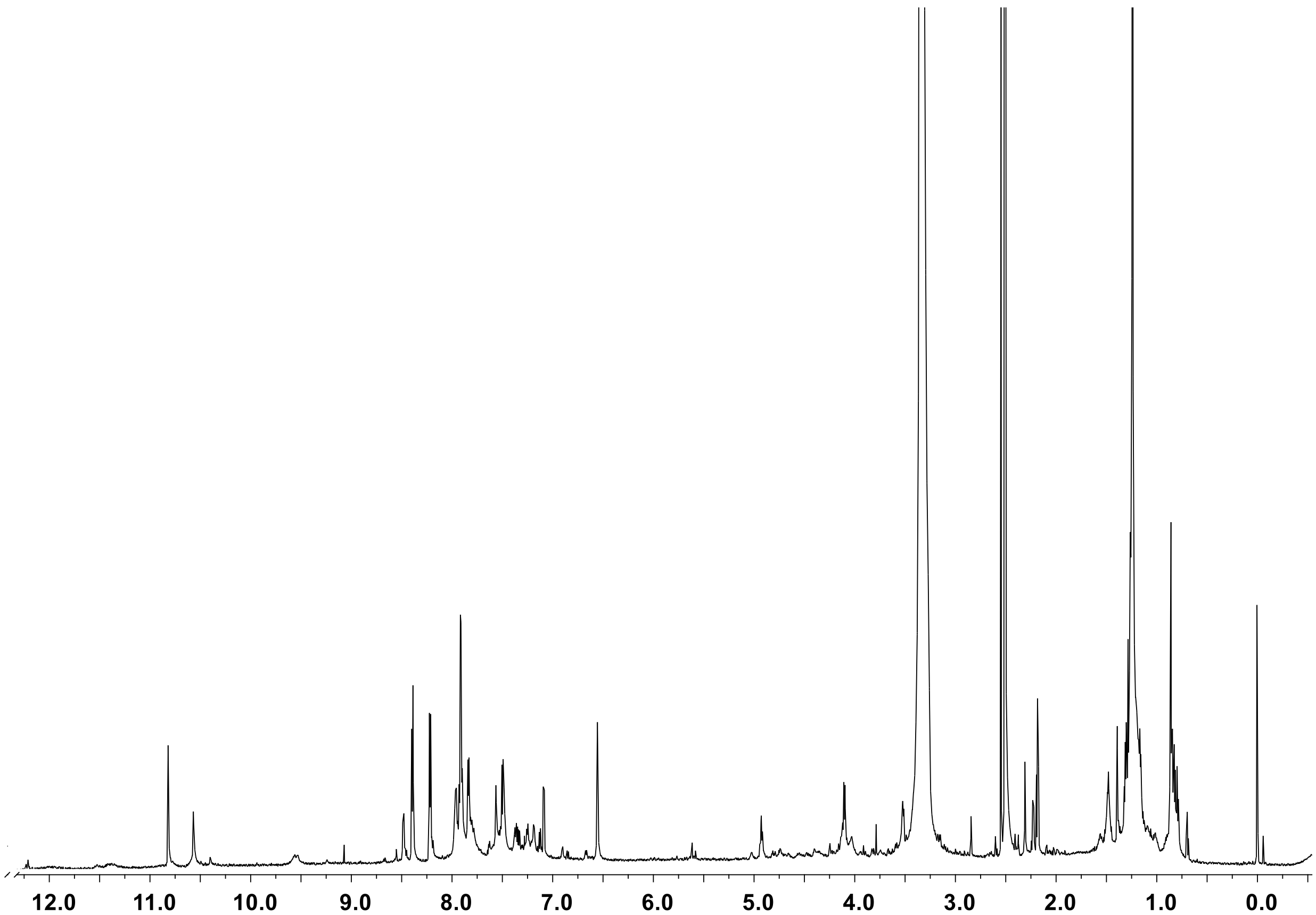

Figure S3. ^{1}H-NMR spectrum of coralmycin J (**1**) measured in DMSO-d_6 at 700 MHz.

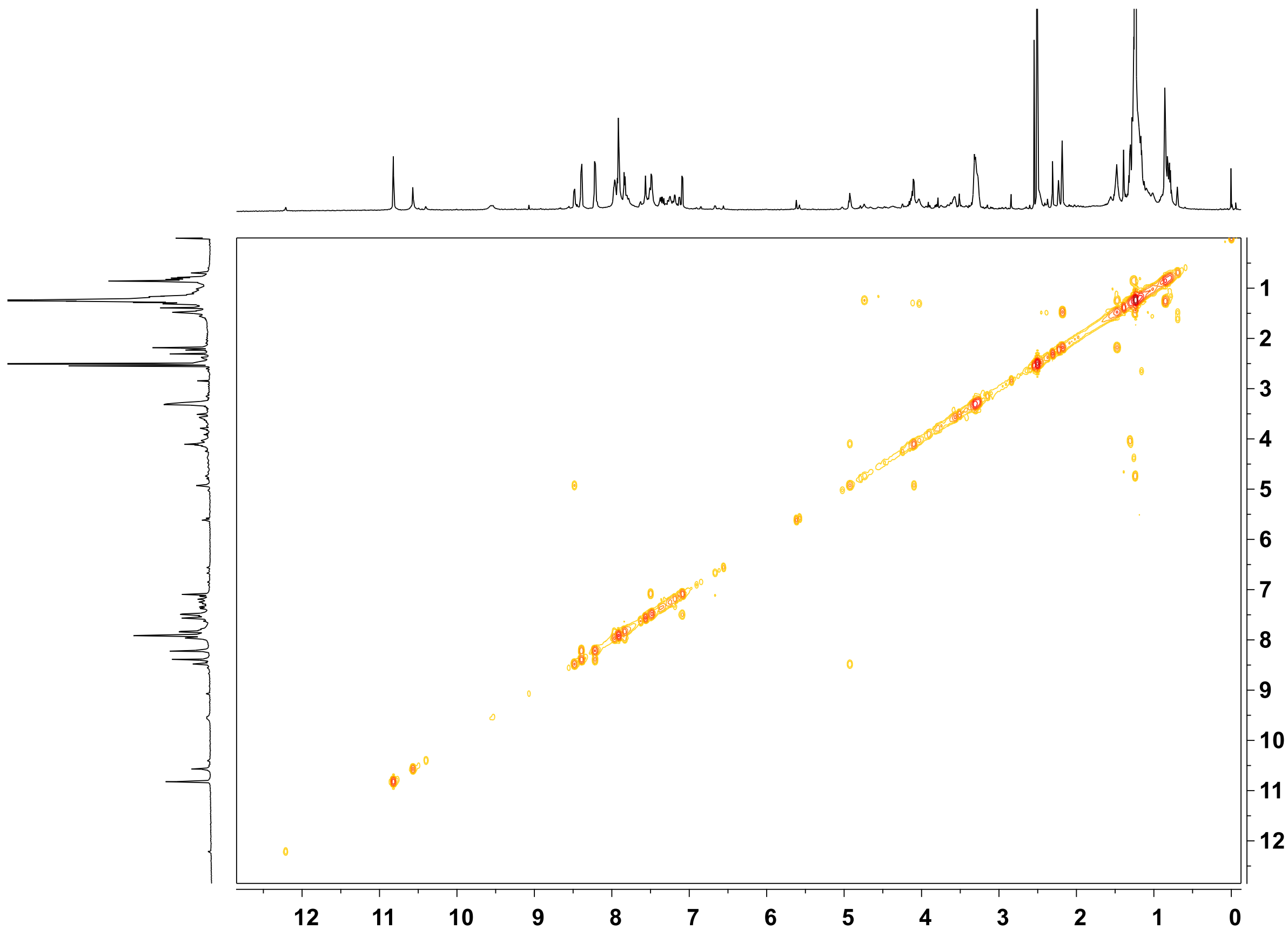

Figure S4. ^{1}H-^{1}H COSY spectrum of coralmycin J (**1**) measured in DMSO-d_6 at 700 MHz.

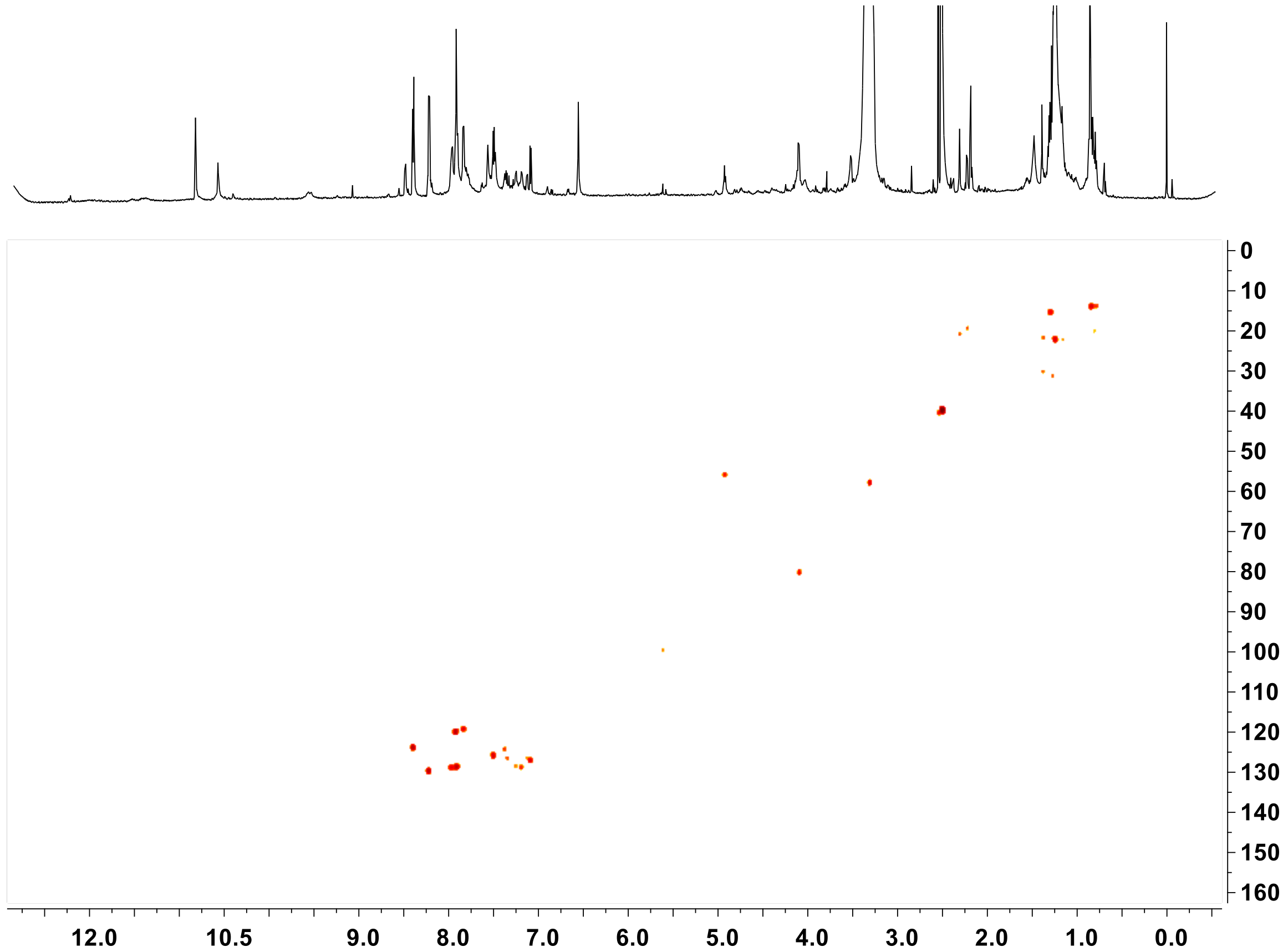

Figure S5. HMQC spectrum of coralmycin J (**1**) measured in DMSO-d_6 at 700 MHz.

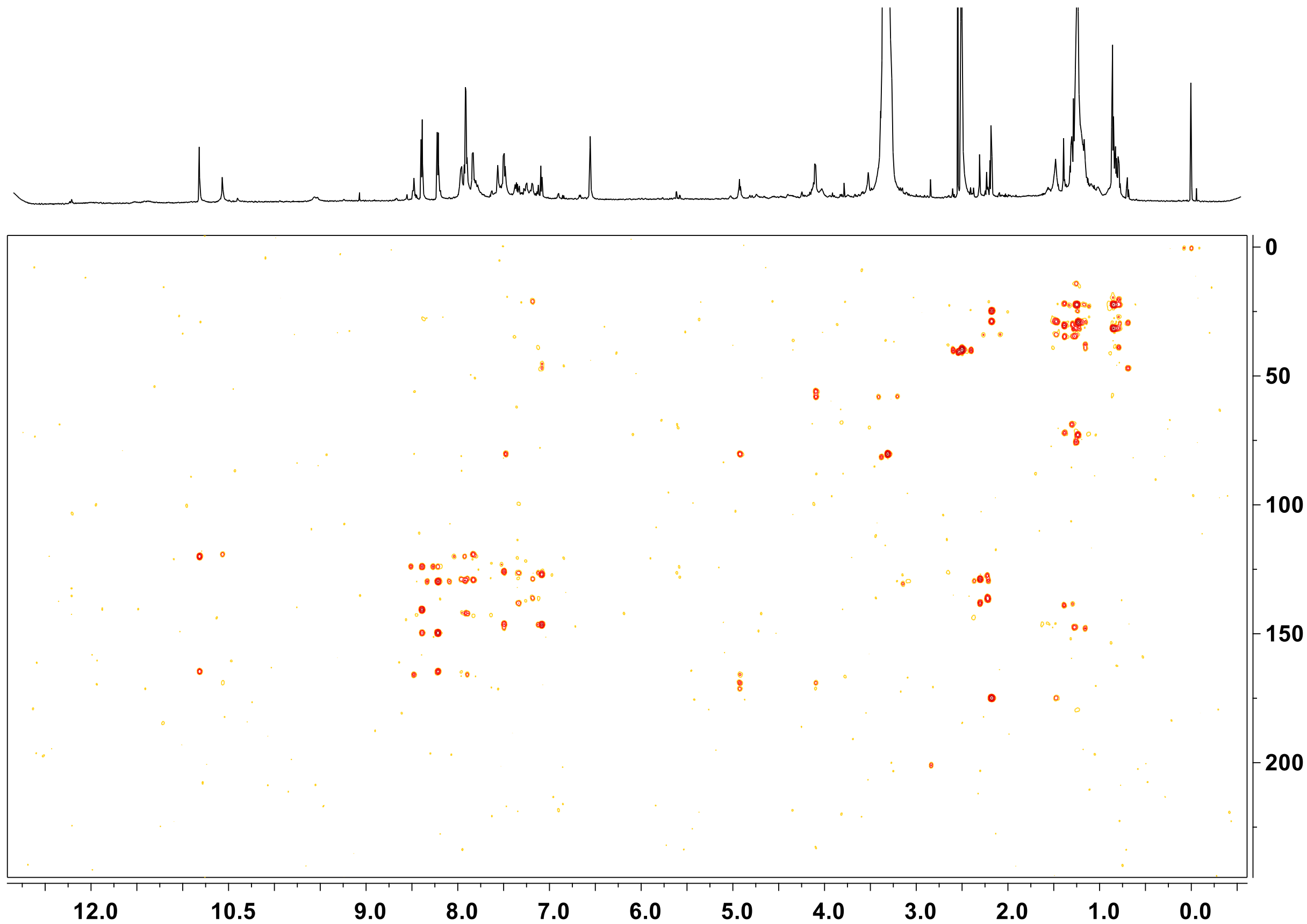

Figure S6. HMBC spectrum of coralmycin J (**1**) measured in DMSO-d_6 at 700 MHz.

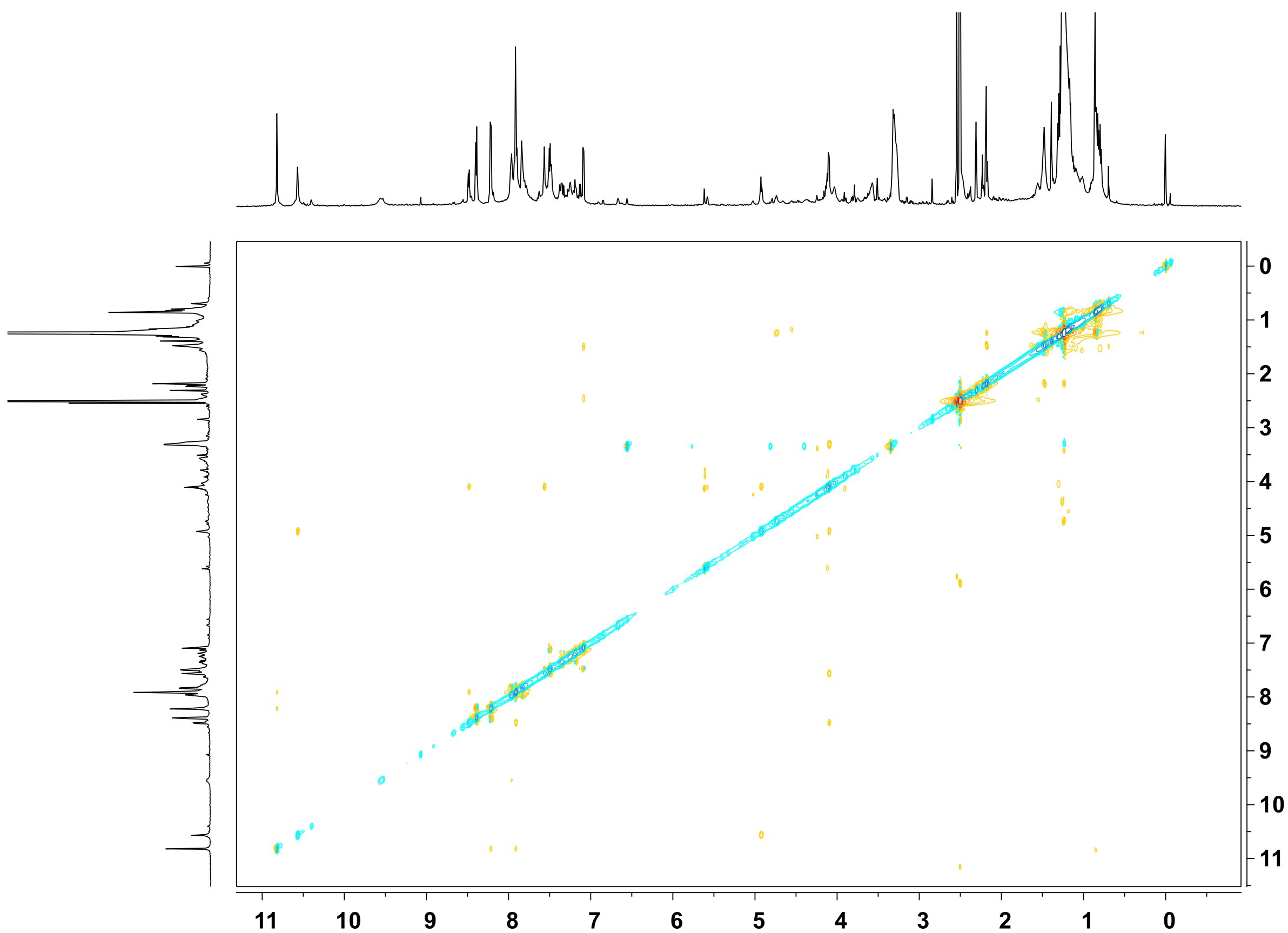

Figure S7. NOESY spectrum of coralmycin J (**1**) measured in DMSO-d_6 at 700 MHz.

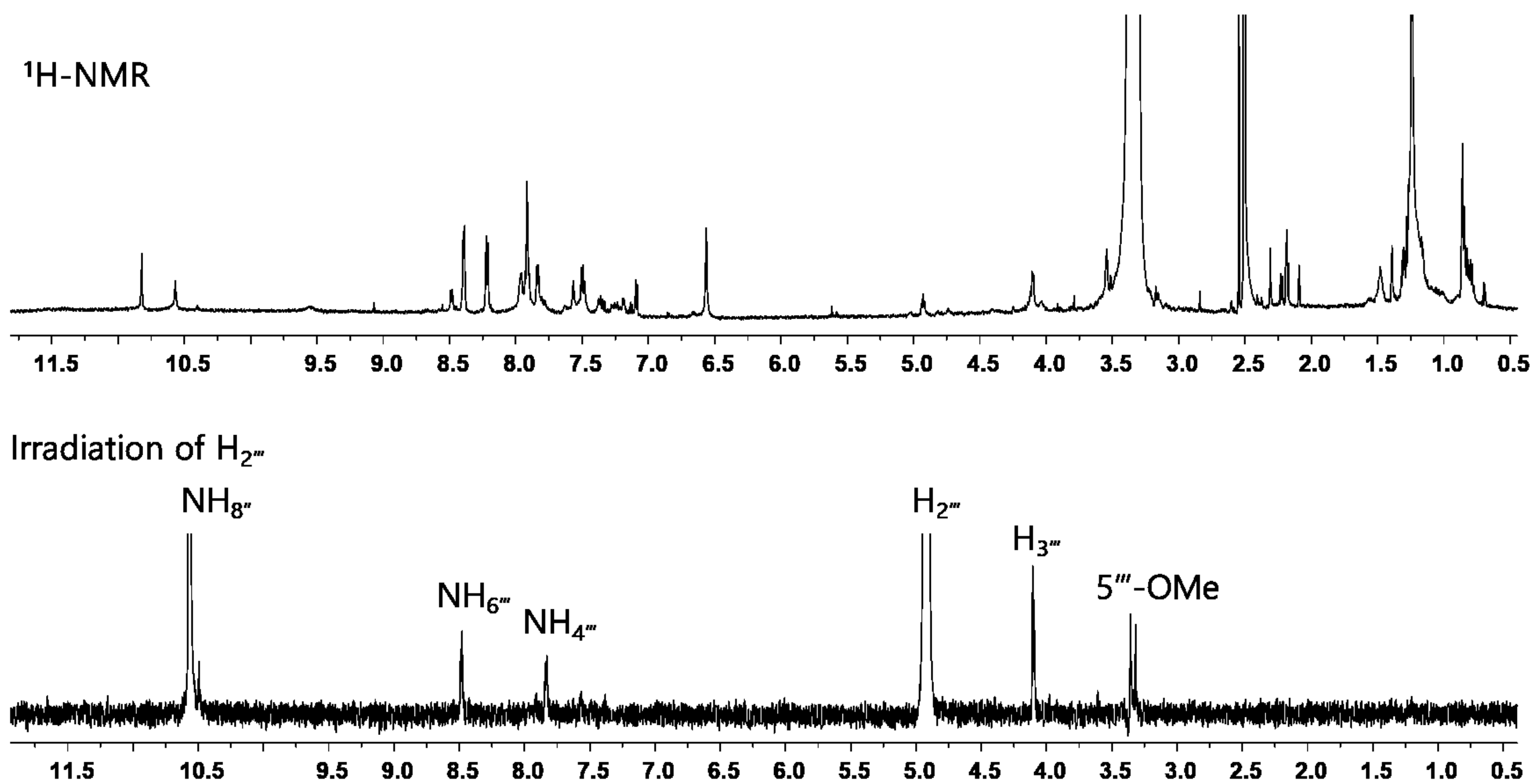

Figure S8. The NOE differential spectra of coralmycin J (**1**) measured in DMSO-d_6 at 700 MHz.

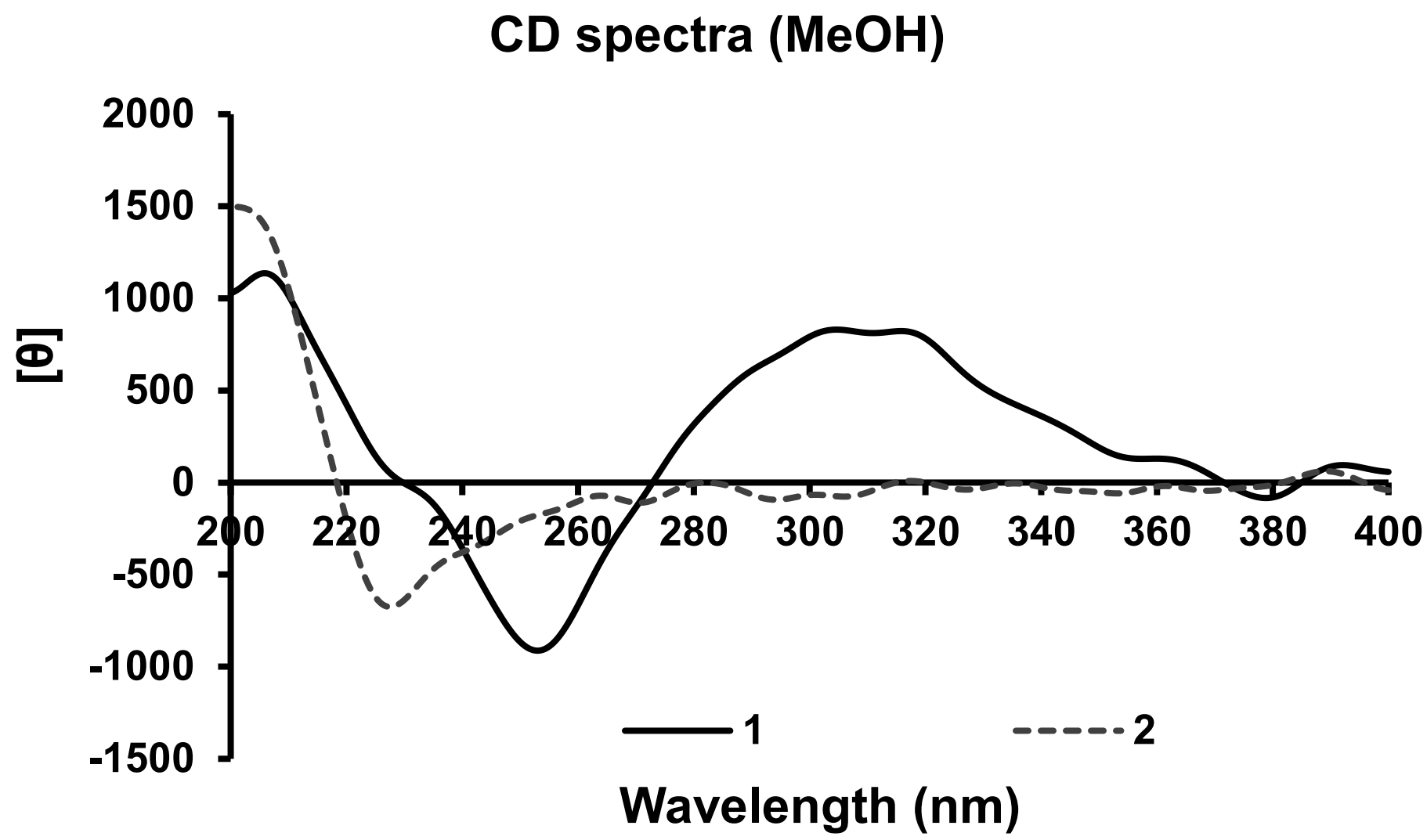

Figure S9. CD spectra of coralmycins J (**1**) and K (**2**)

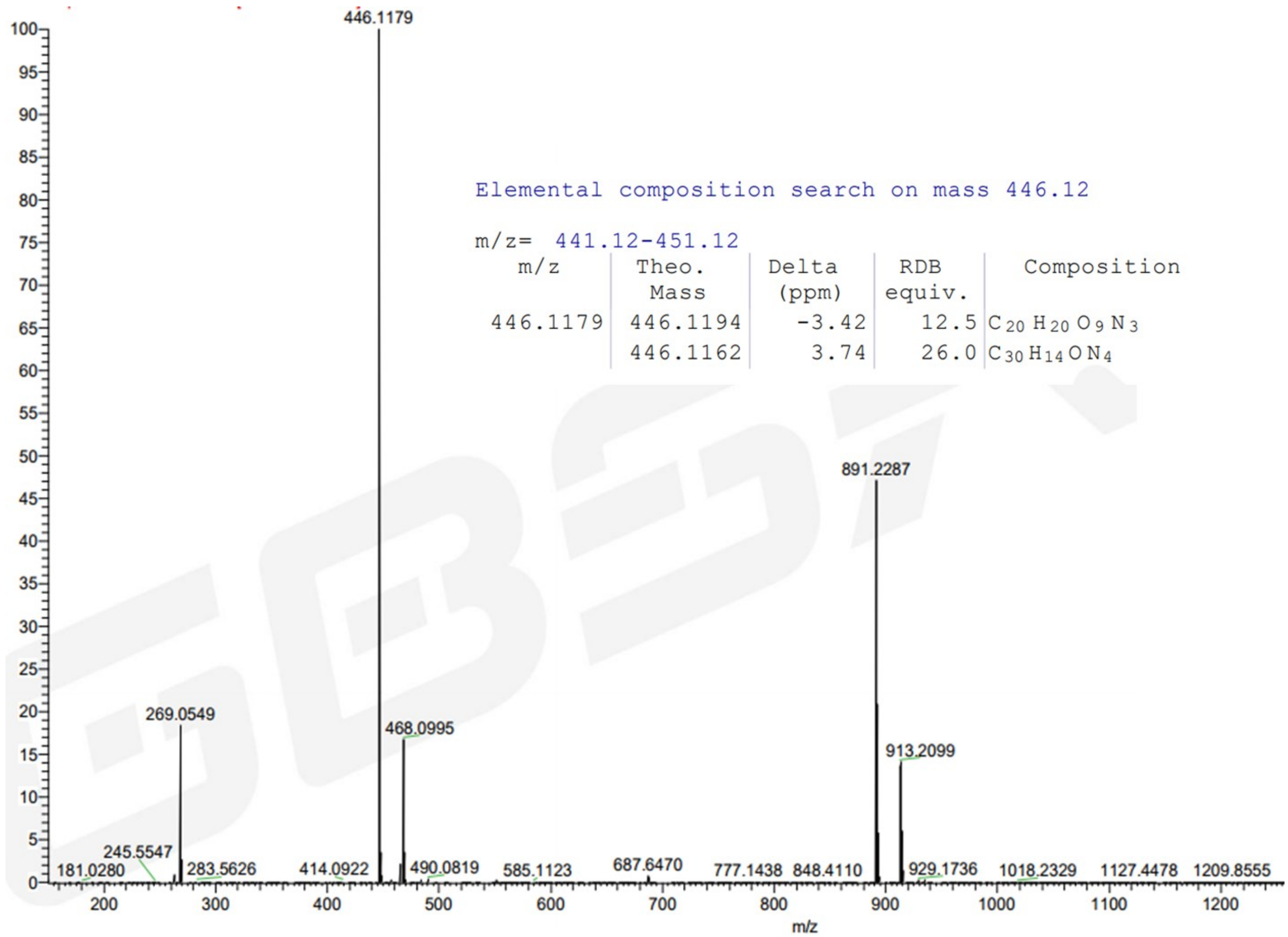

Figure S10. Positive HRESIMS spectrum of coralmycin K (**2**).

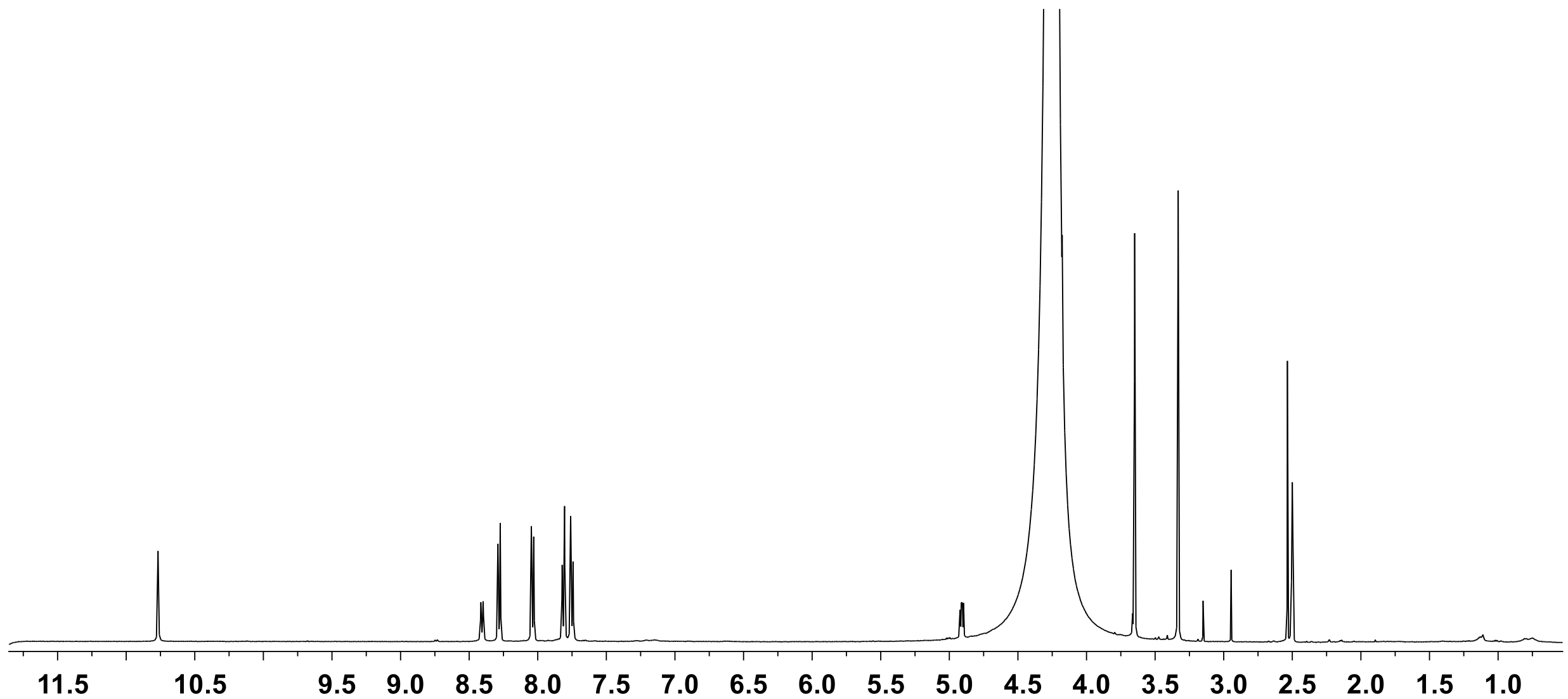

Figure S11. ^{1}H-NMR spectrum of coralmycin K (**2**) measured in DMSO-d_6 at 500 MHz.

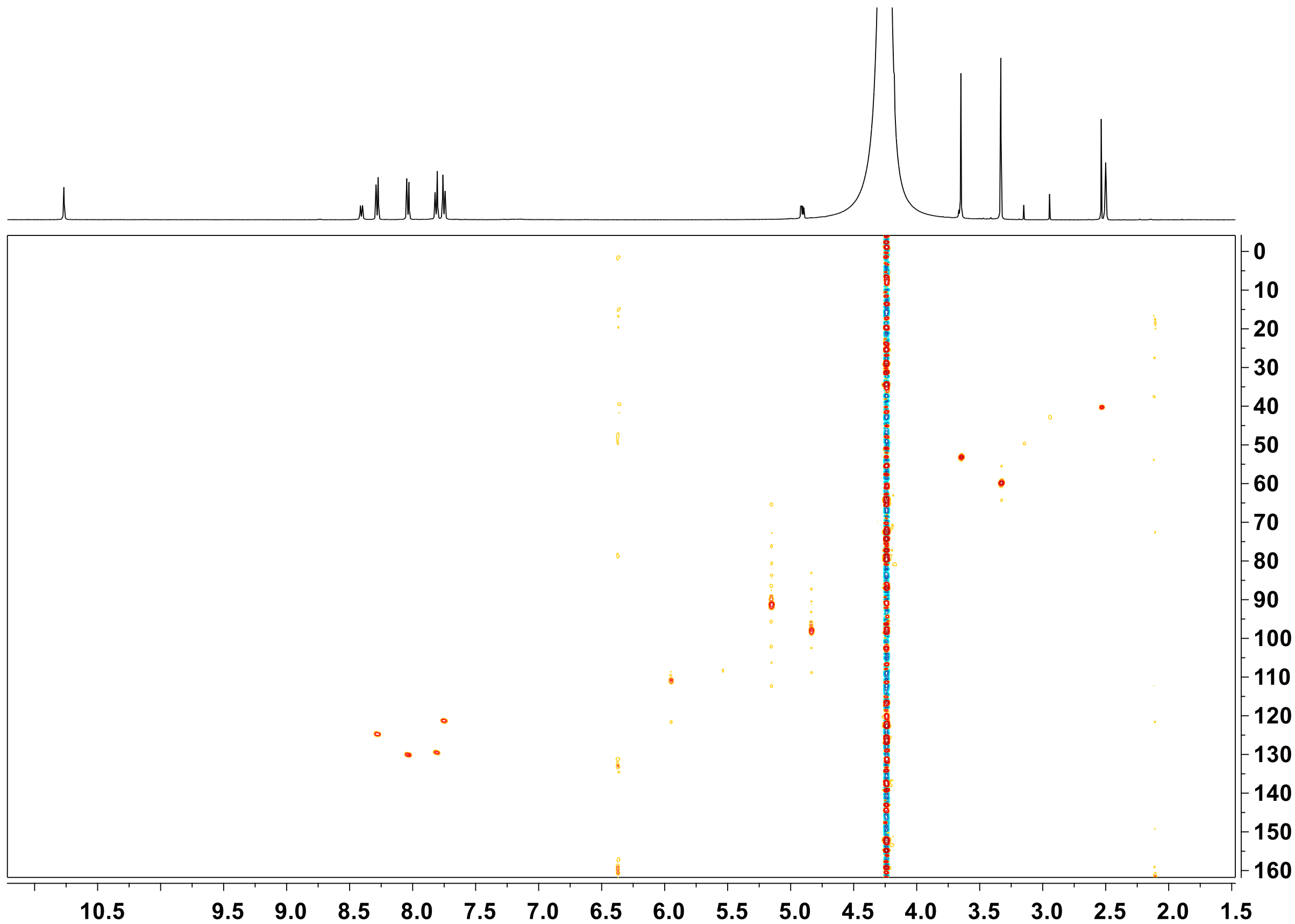

Figure S12. HMQC spectrum of coralmycin K (**2**) measured in DMSO-d_6 at 500 MHz.

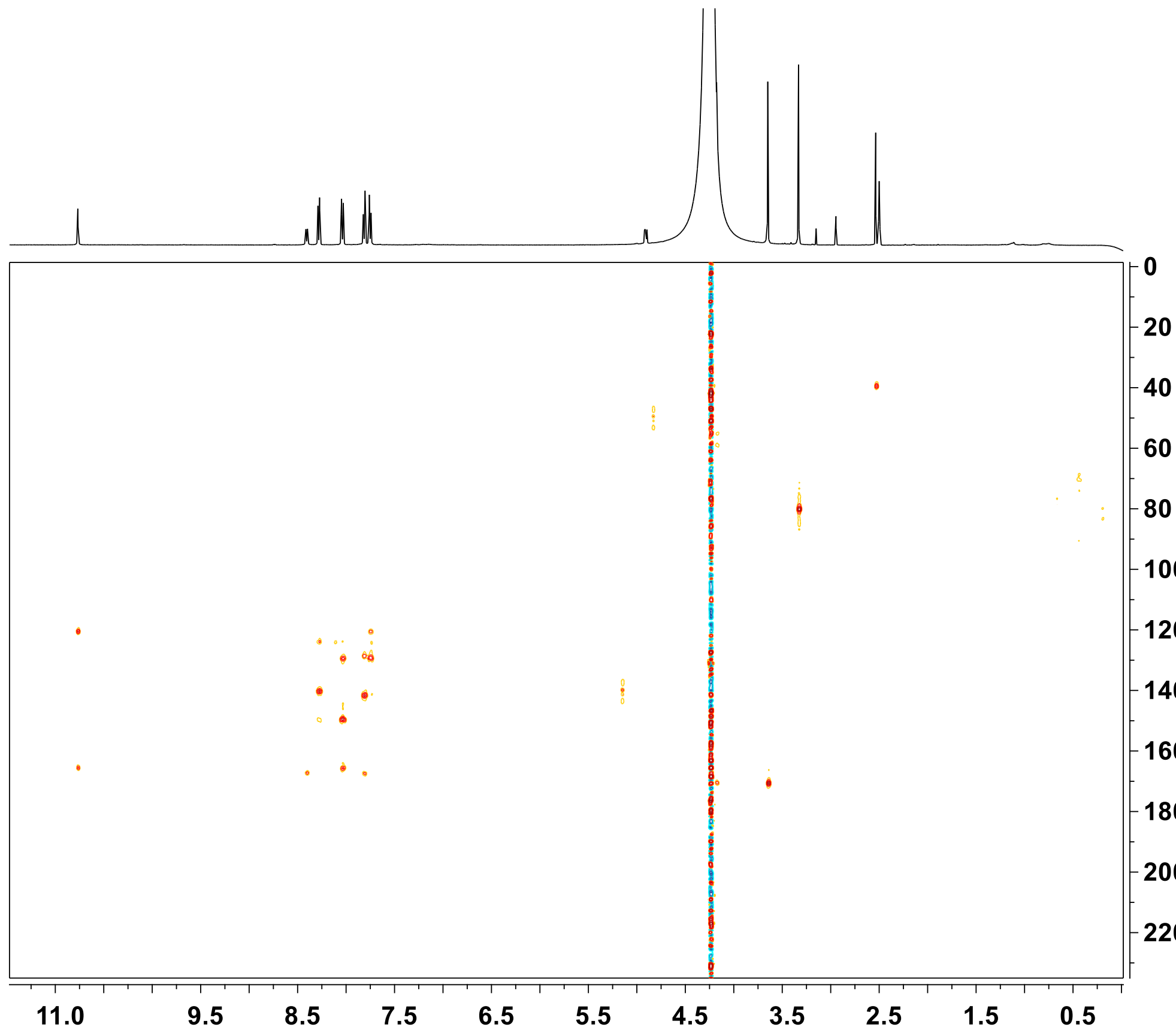

Figure S13. HMBC spectrum of coralmycin K (**2**) measured in DMSO-d_6 at 500 MHz.

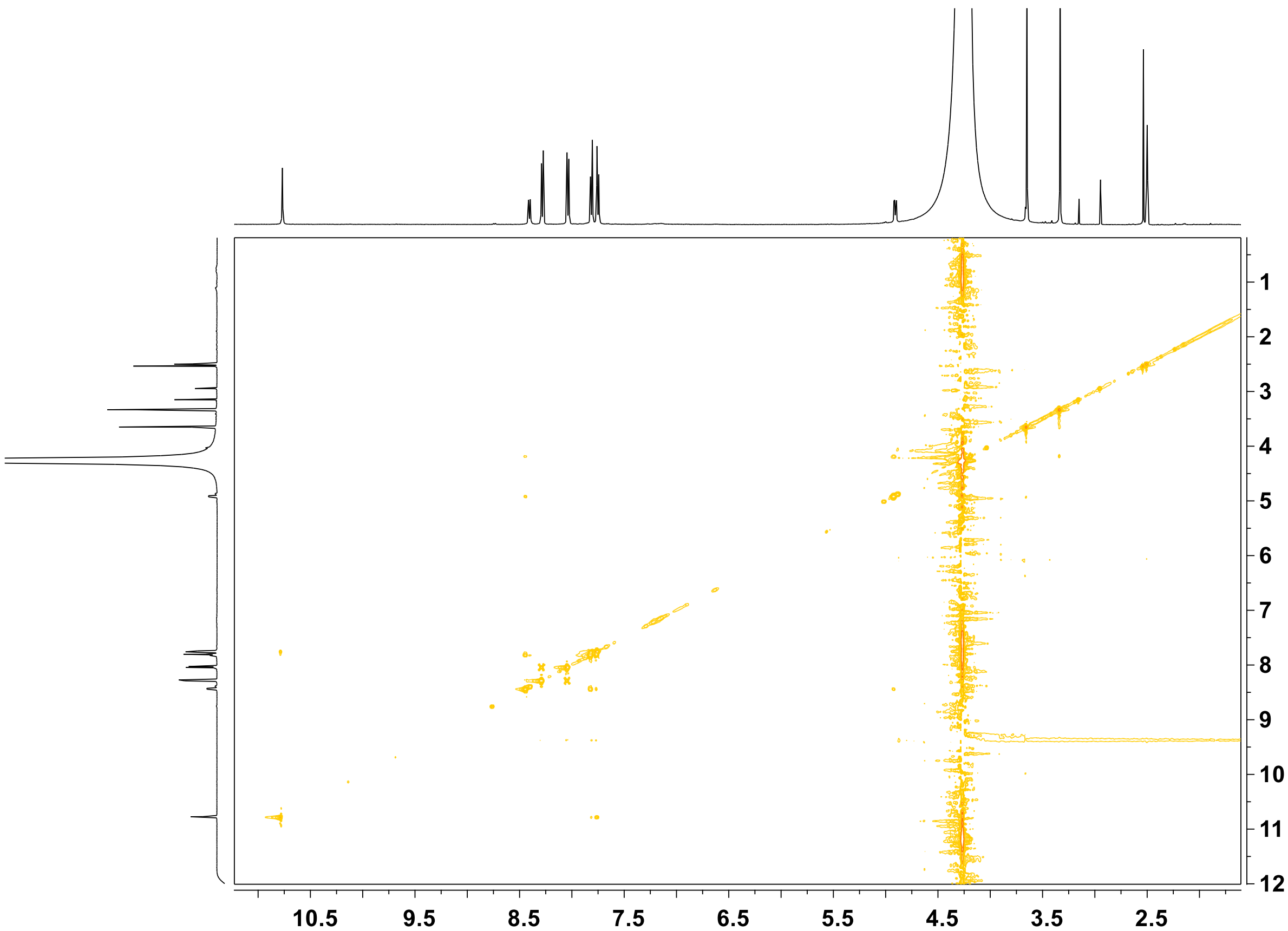

Figure S14. NOESY spectrum of coralmycin K (**2**) measured in DMSO-d_6 at 500 MHz.

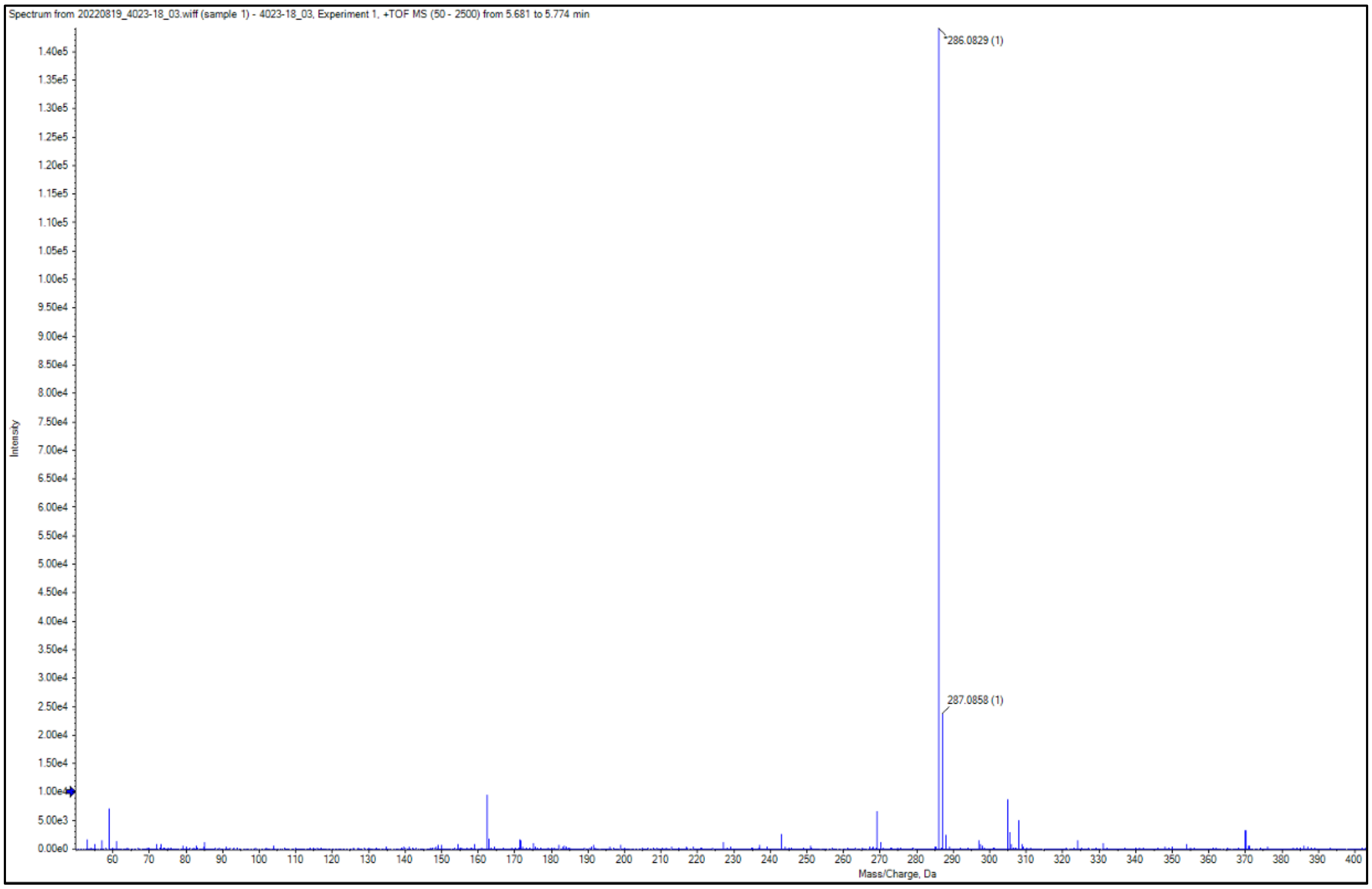

Formula	Charge state	Charged monoisotopic mass	Found at MS
**C14H11N3O4**	**H+**	**286.0822**	**286.0829**

Figure S15. Positive HRESIMS spectrum of coralmycin L (**3**).

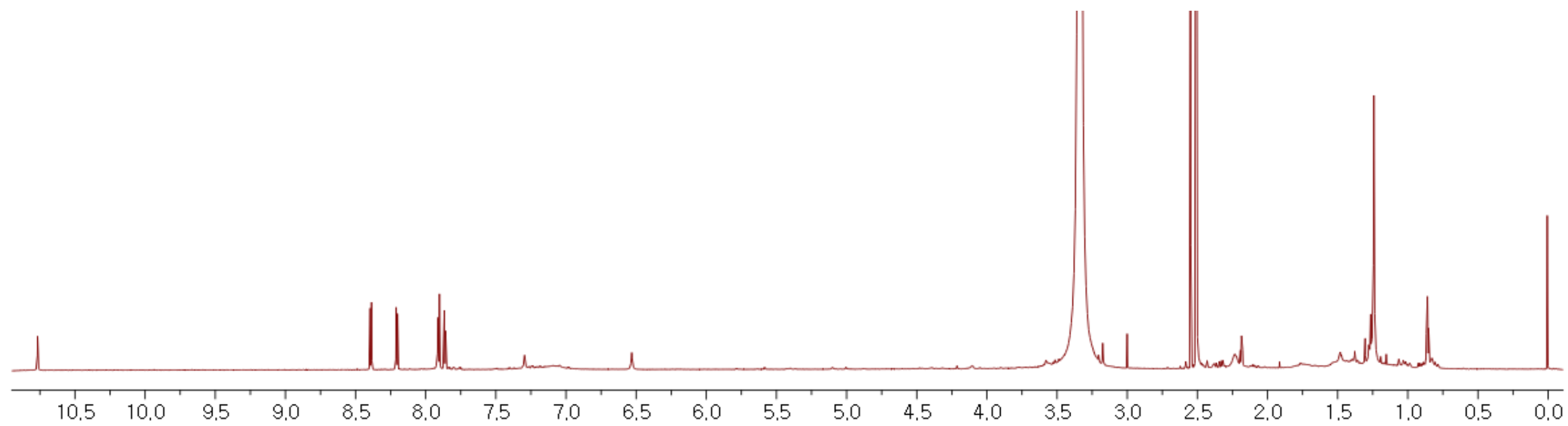

Figure S16. ^{1}H-NMR spectrum of coralmycin L (**3**) measured in DMSO-d_6 at 900 MHz.

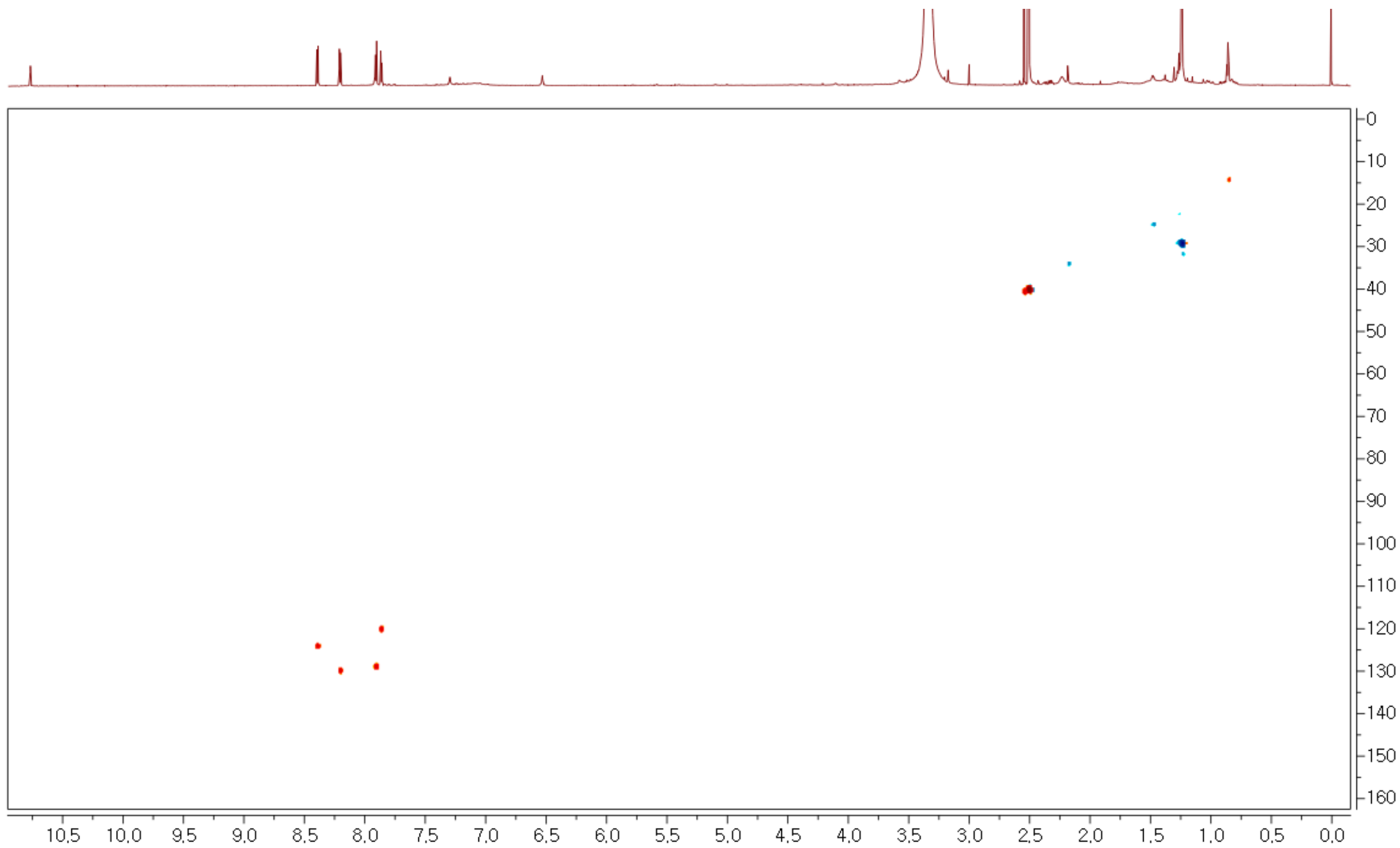

Figure S17. HMQC spectrum of coralmycin L (**3**) measured in DMSO-d$_6$ at 900 MHz.

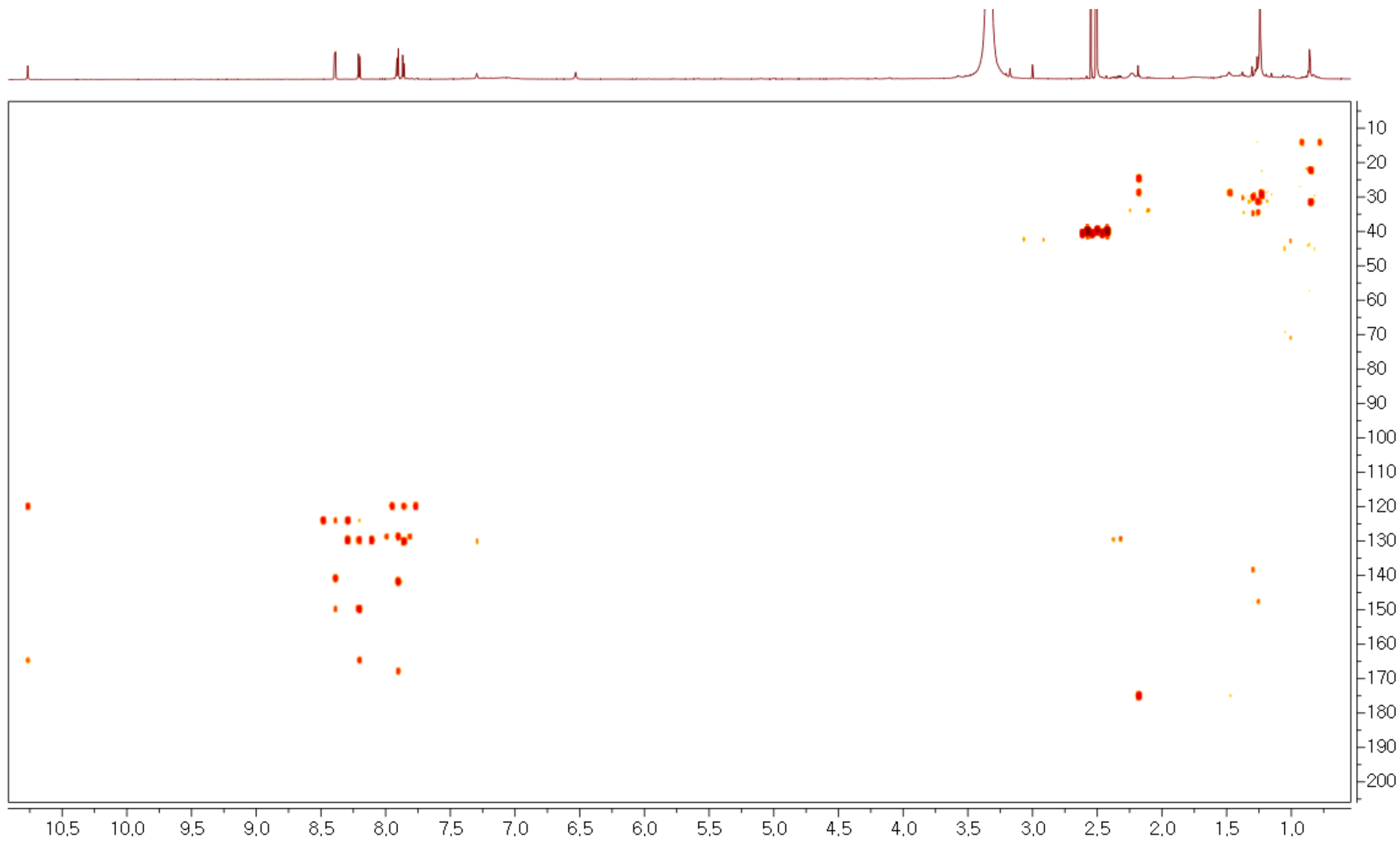

Figure S18. HMBC spectrum of coralmycin L (**3**) measured in DMSO-d$_6$ at 900 MHz.
